# Advances in the Phototriggered Synthesis of Single-Chain Polymer Nanoparticles

**DOI:** 10.3390/polym11111903

**Published:** 2019-11-18

**Authors:** Ester Verde-Sesto, Agustín Blázquez-Martín, José A. Pomposo

**Affiliations:** 1 Centro de Física de Materiales (CSIC, UPV/EHU) and Materials Physics Center MPC, Paseo Manuel de Lardizabal 5, E-20018 San Sebastián, Spain; agustinblazquezmartin@gmail.com (A.B.-M.); josetxo.pomposo@ehu.eus (J.A.P.); 2 Departamento de Física de Materiales, Universidad del País Vasco (UPV/EHU), Apartado 1072, E-20800 San Sebastián, Spain; 3 IKERBASQUE – Basque Foundation for Science, María Díaz de Haro 3, E-48013 Bilbao, Spain

**Keywords:** photochemistry, photofolding, single-chain nanoparticles

## Abstract

Clean use of photons from light to activate chemical reactions offers many possibilities in different fields, from chemistry and biology to materials science and medicine. This review article describes the advances carried out in last decades toward the phototriggered synthesis of single-chain polymer nanoparticles (SCNPs) as soft nanomaterials with promising applications in enzyme-mimicking catalysis and nanomedicine, among other different uses. First, we summarize some different strategies developed to synthesize SCNPs based on photoactivated intrachain homocoupling, phototriggered intrachain heterocoupling and photogenerated collapse induced by an external cross-linker. Next, we comprehensively review the emergent topic of photoactivated multifolding applied to SCNP construction. Finally, we conclude by summarizing recent strategies towards phototriggered disassembly of SCNPs.

## 1. Introduction

Light from the sun is one of the cheapest and most available energy sources in the Earth. Moreover, photons from sunlight can activate many chemical reactions. As early as 1908, photochemistry pioneer Giacomo Ciamician recognized the milder reaction conditions of photoactivated reactions when compared to thermal procedures [1,2]. At these early dates, Ciamician was able to envision photochemistry as an artificial way to put solar energy into practical uses [3].

**Figure 1:**
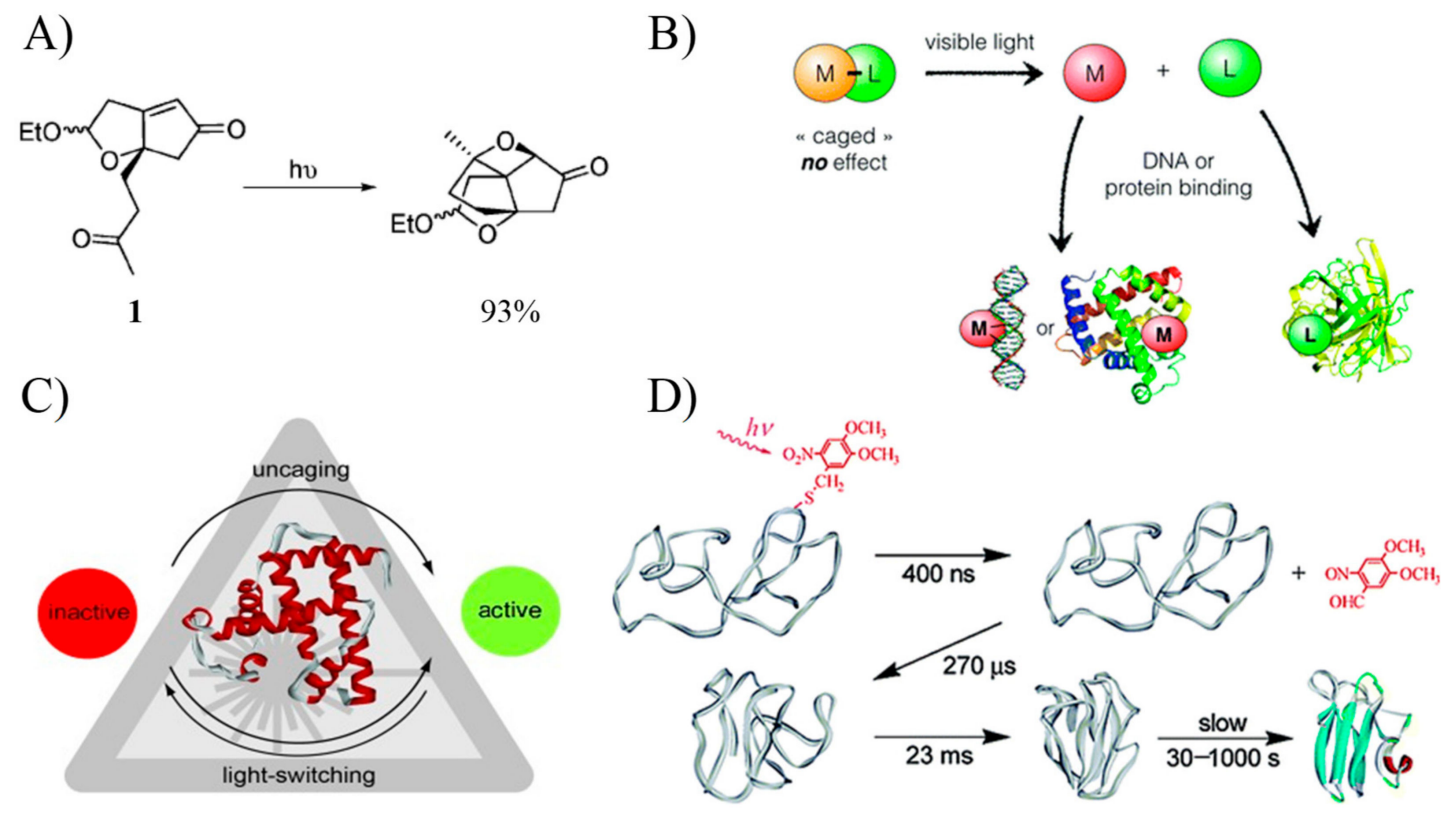
(**A**) An illustration of the use of photochemistry for easy construction of the complex tetracyclic ring of merrilactone A, a potent neurotrophic compound isolated from the pericarps of the *Illicium merrillianum* plant, starting from compound **1**. (**B**) The principle of photoactivated chemotherapy using a non-toxic metal–ligand (M–L) compound: upon photocleavage of the M–L bond, either the fragment M or L, or both, display toxicity to cancer cells. (**C**) The use of photoresponsive structural cross-linkers allows phototriggering to be employed as a useful method to investigate proteins’ conformational changes. (**D**) The introduction of photolabile motifs enables the investigation of initial protein folding dynamics. Reprinted from [10,14,15,16] with permission.

In general, the energy of photons in the ultraviolet (UV) and visible regions of the electromagnetic spectrum is enough to promote molecules to excited electronic states [4]. Upon photoactivation, often challenging transformations result under very mild conditions without the use of high temperatures or aggressive reagents. Some uses of photochemistry include phototriggered oxidations [5], isomerizations [6], cross-couplings [7], deprotections [8] and cycloadditions [9]. As an example, Figure 1A illustrates the power of photochemical [2 + 2] cyclizations for constructing complex tetracyclic rings, as found in some natural compounds. Hence, a simple intramolecular [2 + 2] Paternò–Büchi photoaddition from keto-enone **1** (see structure in Figure 1A) produces, in high yield [10], the central oxetane ring of merrilactone A, a potent neurotrophic compound isolated from the pericarps of *Illicium merrillianum* plant.

Concerning biochemistry, Engels [11] and Kaplan [12], in the late 1970s, pioneered the UV-induced deprotection of “photocaged” nucleotides, such as cyclic adenosine monophosphate (cAMP) and adenosine triphosphate (ATP). Hence, through protection of the phosphate group of cAMP and ATP with a photocleavable nitrobenzyl group they were able to shown that the biological function of these molecules recovered after cleavage via UV irradiation. Morrison, in the late 1980s, pioneered the idea of photoactivation in inorganic chemistry by introducing the compound Rh(phen)_2_Cl_2_^+^ which upon UV irradiation was able to bind DNA irreversibly [13]. Modern photoactivated chemo- therapeutic (PACT) compounds follow this concept: the non-irradiated form of the metal–ligand (M–L) compound has no biological activity and low toxicity, while the irradiated form has high toxicity to cancer cells by interacting with DNA, lipids or proteins (see Figure 1B) [14]. In biology, the fundamental principles that govern protein folding, misfolding and aggregation have often been explored by the introduction of photoresponsive structural cross-linkers (Figure 1C) [15] and photolabile motifs (Figure 1D) [16].

Concerning polymer chemistry, photochemical reactions facilitate precision polymer design allowing easy end-group and polymer functionalization, as well as surface modification and cross-linking [17]. As ultra-fine cross-linked nano-objects, single-chain polymer nanoparticles (SCNPs) obtained via folding/collapse of individual linear polymer chains have attracted significant interest in recent years due to their favorable prospects for catalysis, drug delivery and sensing applications [18]. Single chain technology allows constructing bioinspired, ultra-small (3–30 nm) SCNPs via intra-chain cross-linking at high dilution conditions. Both covalent and non-covalent bonds have been employed to produce SCNPs. On one hand, the use of non-covalent interactions leads to adaptive nano-objects, which can respond to different stimuli. On the other hand, covalent bonds give rise to robust SCNPs with enhanced stability against temperature, pH, sonication, etc. More importantly, the resulting SCNPs are often endowed with local compact domains (local pockets) to which drugs or catalyst could be either temporally or permanently immobilized, paving the way to the development of innovative drug delivery systems or enzyme-mimetic catalysts, among other potential applications [18]. The interest in the use of photochemistry to synthesize SCNPs has also grown in parallel to the development of potential applications. In fact, the advantages of photoactivated synthesis of SCNPs include: (i) relatively mild reaction conditions, (ii) the possibility to investigate the complex SCNP formation process via phototriggered multifolding and (iii) in the case of some photocoupling reactions, the ability to endow the SCNPs with fluorescence properties.

**Scheme 1:**
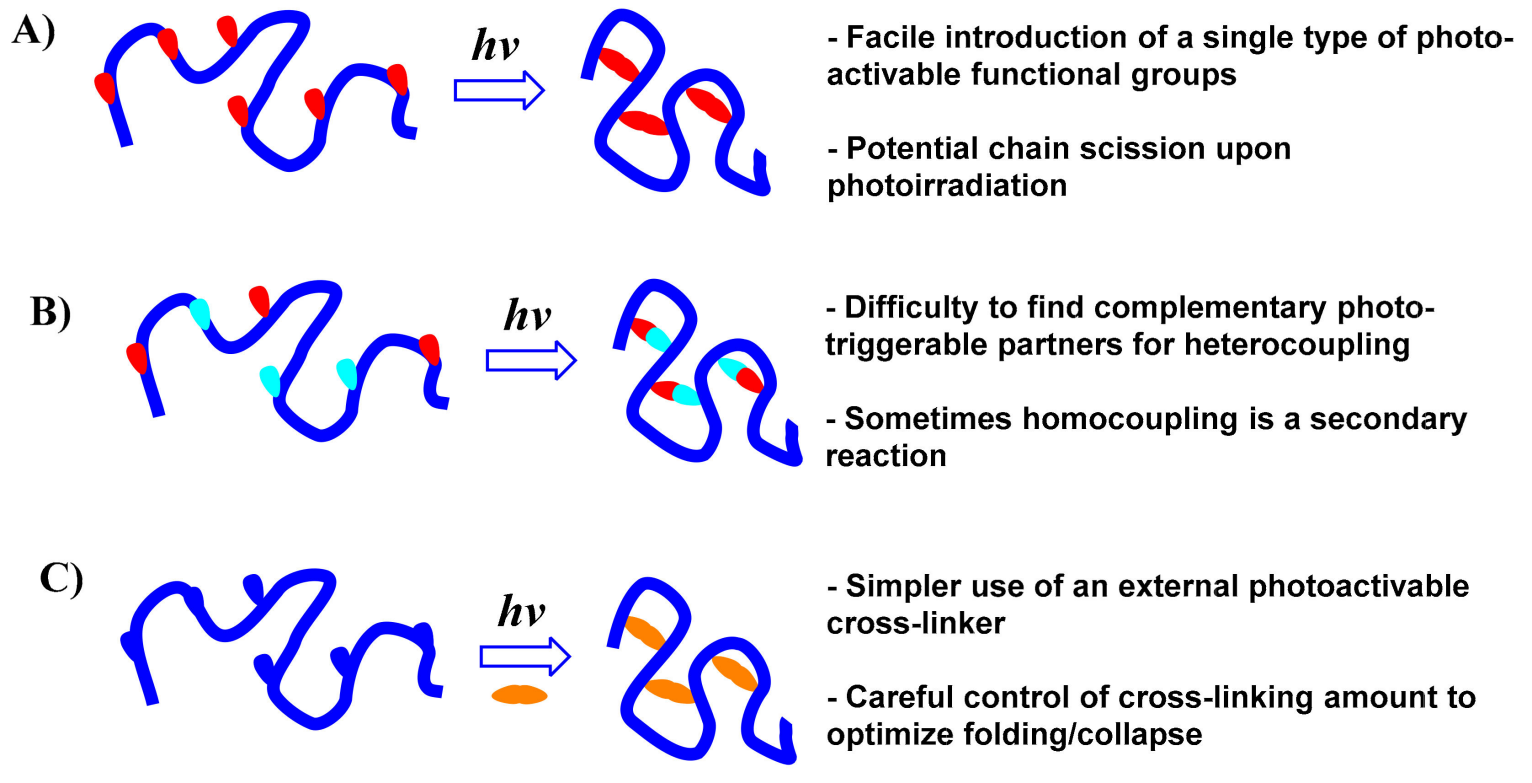
Schematic illustration of the different strategies developed to synthesize single-chain polymer nanoparticles (SCNPs) based on photochemical reactions and their advantages/limitations: (**A**) Photoactivated intrachain homocoupling. (**B**) Phototriggered intrachain heterocoupling. (**C**) Photogenerated collapse induced by an external cross-linker.

In this review article, we describe the different strategies developed to synthesize SCNPs based on photochemical reactions (see Scheme 1), including methods such as (i) photoactivated intrachain homocoupling, (ii) phototriggered intrachain heterocoupling and (iii) photogenerated collapse induced by external cross-linkers. Additionally, we comprehensively review the emergent branch of photoactivated multifolding applied to SCNP construction. To conclude, we cover recent strategies towards the phototriggered disassembly of SCNPs.

## 2. Synthesis of Single-Chain Polymer Nanoparticles via Photoactivated Intrachain Homocoupling

In a broad sense, photoactivated intrachain homocoupling refers to the folding/collapse process induced by photons to an isolated polymer chain through the involvement of a single type of photoresponsive functional group (see Scheme 1A). Nowadays, the different strategies developed to synthesize SCNPs based on photoactivated intrachain homocoupling include: (i) photodimerization, (ii) photocyclization, (iii) photoisomerization, (iv) photogeneration of reactive species and (iv) self-assembly induced by photodeprotection.

### 2.1. Photodimerization

**Figure 2:**
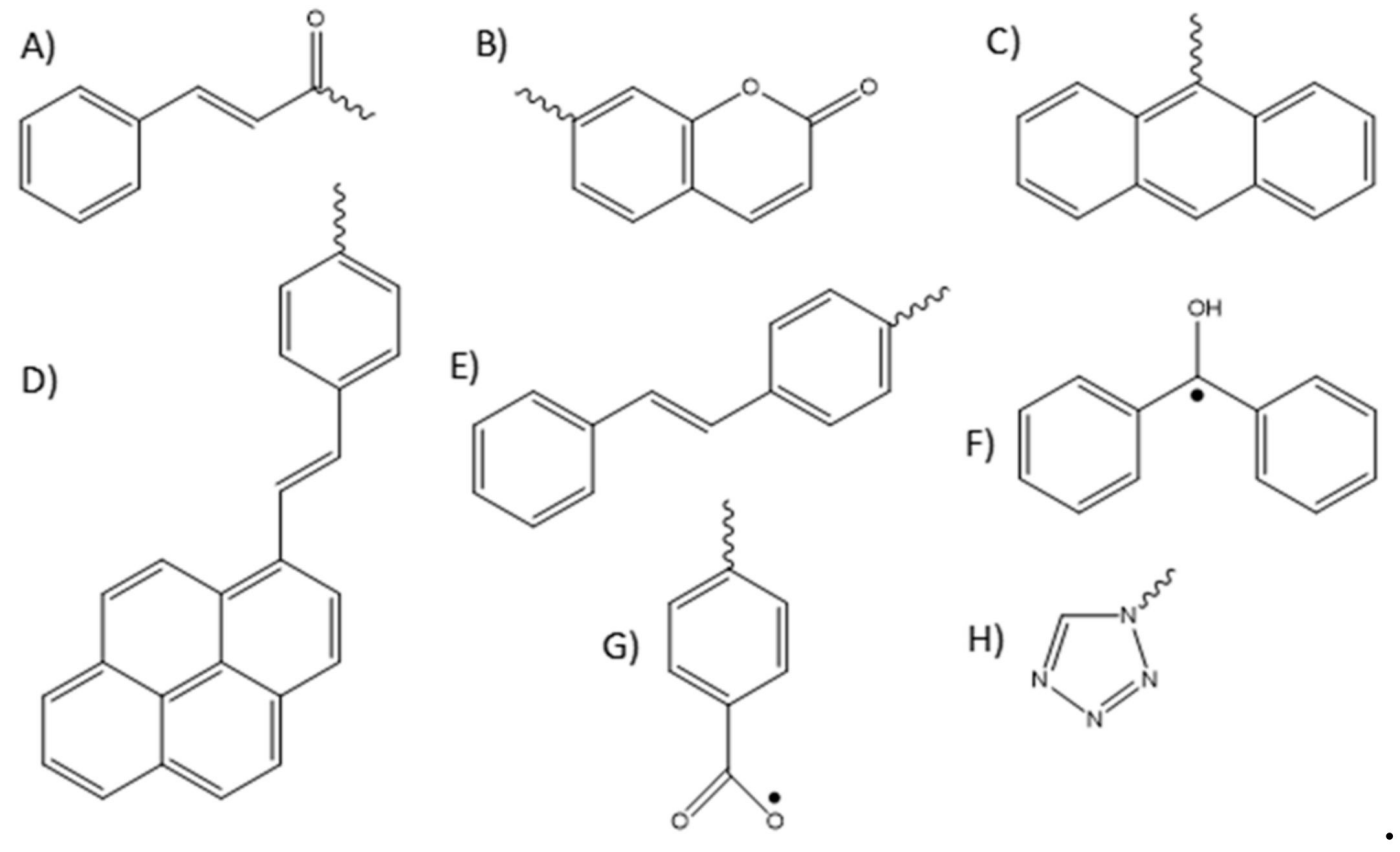
Functional groups employed for SCNP generation via intrachain photodimerization: (**A**) cinnamoyl, (**B**) coumarin, (**C**) anthracene, (**D**) styrylpyrene, (**E**) stilbene, (**F**) ketyl radical, (**G**) carboxyl radical and (**H**) nitrile imine (for details see text below).

Figure 2 shows the main functional groups used in photodimerization for SCNP formation via photoactivated intrachain homocoupling.

#### 2.1.1. Cinnamoyl Functional Groups

Photodimerization of cinnamoyl units (see Figure 2a) was one of the earliest photochemical reactions adopted to produce SCNPs. Hence, Tao and Liu in the late 1990s pioneered the UV-induced formation of tadpole-shaped SCNPs using diblock copolymers in which one of the blocks contained photodimerizable cinnamoyl moieties and the other contained inert styrene repeat units [19]. UV irradiation of unimer chains of the precursor diblock copolymer produced tadpole-shaped SCNPs via intrachain cinnamoyl dimerization, as confirmed by combining UV absorbance analysis, size exclusion chromatography (SEC), static light scattering (SLS) and nuclear magnetic resonance (NMR) spectroscopy measurements. In a further work by this group, the photochemical technique was refined to obtain essentially pure, tadpole-shaped SCNPs at a high final polymer concentration [20]. Imprinted, tadpole-shaped SCNPs of 7.4 nm in diameter with exceptionally a high binding capacity and high selectivity for l-phenylalanine anilide were fabricated by means of this improved photoactivated synthesis technique [21]. Very recently, cinnamoyl dimerization has been used by He and coworkers [22] to investigate the self-assembly of amphiphilic diblock copolymers with the photocross-linkable units placed either in the hydrophobic or the hydrophilic blocks. When compared to the unreacted diblock copolymers, significant differences were found for the resulting tadpole-shaped SCNPs. Hence, photoinduced collapse of the hydrophobic block induced a morphological transition from branched cylindrical micelles to completely spherical micelles at a cinnamoyl dimerization degree of ca. 63%. When photocross-linking was performed in the hydrophilic block, the sizes of the micelles showed a dramatic increase due to a shift of the hydrophobic/hydrophilic balance. Interestingly, at a dimerization degree > 60% the tadpole-shaped SCNPs assembled into non-conventional aggregates with non-smooth surfaces.

#### 2.1.2. Coumarin Functional Groups

**Figure 3:**
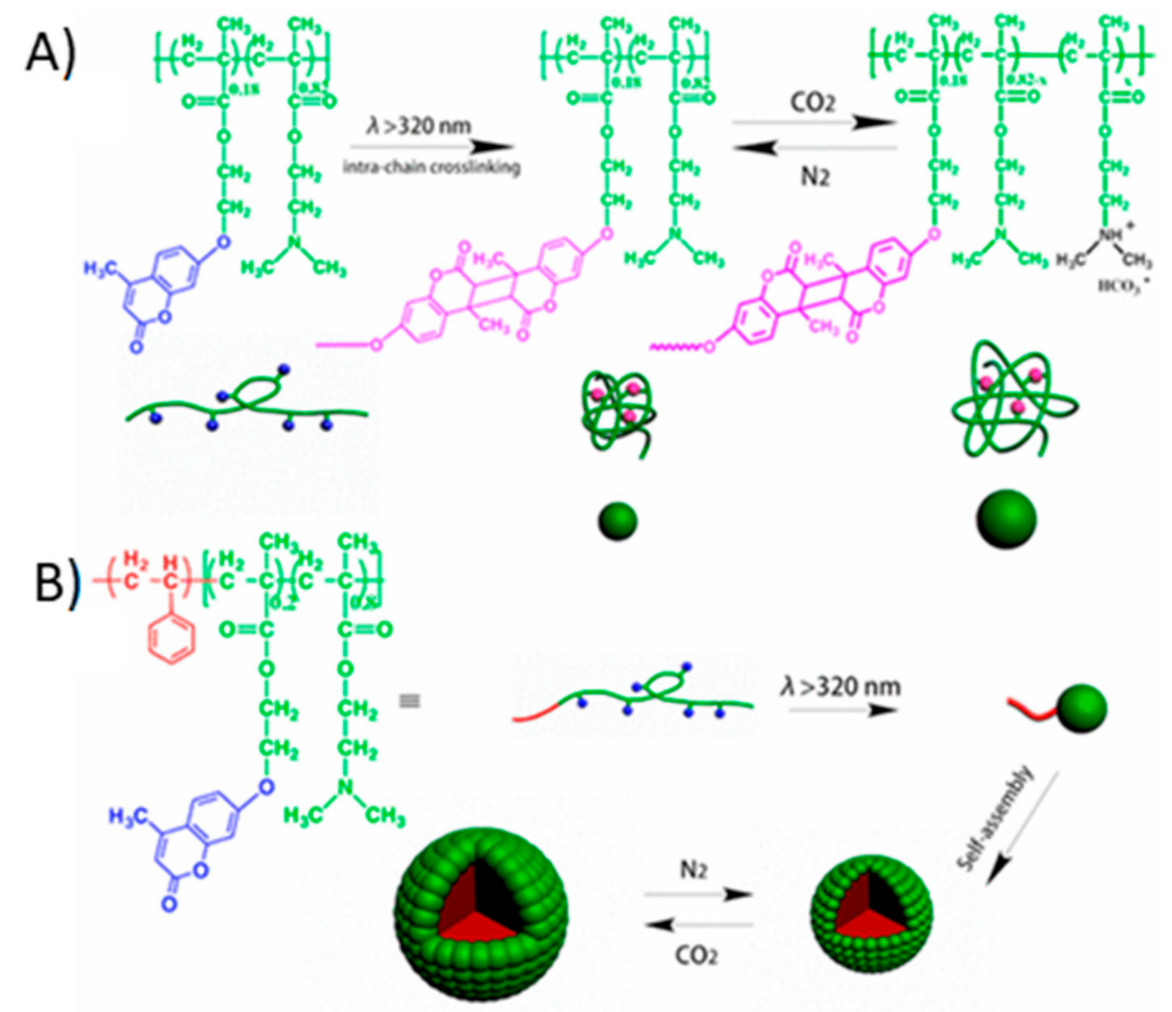
Schematic illustration of the synthesis via intrachain coumarin dimerization of: (**A**) CO_2_-responsive SCNPs, and (**B**) CO_2_-responsive micelles self-assembled from amphiphilic tadpole-shaped Janus SCNPs. Reprinted from [24] with permission.

Zhao and coworkers reported in 2011 the preparation of neat SCNPs using the intramolecular photodimerization of coumarin moieties (Figure 2b) upon λ > 310 nm UV irradiation in a dilute solution of coumarin-containing copolymers (7 or 13 mol % of coumarin units) [23]. The dimerization degree was ca. 75% after 1 h irradiation, as determined by UV absorbance analysis. The resulting SCNPs were characterized by SEC, solution viscosity, differential scanning calorimetry (DSC) and ^1^H NMR spin–spin relaxation time measurements. These SCNPs were further used as useful nanoreactors to synthesize gold nanoparticles (AuNPs). Remarkably, the rate of AuNP formation was found to depend on the dimerization degree of the coumarin moieties, which significantly determined the SCNP compaction degree and presumably also the intrachain mobility. Attempts to disassemble the SCNPs via photocleavage of the coumarin dimers under UV irradiation at λ < 260 nm were unsuccessful, as revealed by SEC experiments. In fact, after 2 h of irradiation, the dimerization degree remained as high as 38%. In a further work by this group [24], CO_2_-responsive SCNPs and micelles self-assembled from amphiphilic, tadpole-shaped Janus SCNPs were prepared, relying on the intramolecular photodimerization of coumarin units from diblock copolymers (see Figure 3). Both the dimensions of the SCNPs and those of self-assembled micelles from tadpole-shaped Janus SCNPs were found to undergo reversible swelling/shrinking in response to CO_2_/N_2_ bubbling. Hence, CO_2_/N_2_ stimulation was investigated by these authors to develop gas-tunable nanoreactors for synthesizing AuNPs, in which both the kinetics of AuNP formation and the AuNP size can be finely controlled. Subsequently, Zhao and coworkers reported photoresponsive liquid crystalline single chain nanoparticles (LC-SCNPs) prepared via intramolecular photodimerization of coumarin using a side-chain liquid crystalline polymer containing coumarin moieties and azobenzene mesogens [25]. Interestingly, the resulting LC-SCNPs exhibited several interesting properties: (i) when dispersed in chloroform (poor solvent), they displayed a fluorescent emission that was dependent on the cis/trans ratio of azobenzene mesogens; (ii) when exposed to linearly polarized irradiation, they underwent photoinduced deformation that was dependent on the excitation wavelength; and (iii) when dispersed in a poly(methyl methacrylate) (PMMA) matrix to form a nanocomposite film that was stretched at 113 °C to 400% strain followed by cooling to room temperature, the stretched film displayed strong parallel dichroism as a consequence of the stretching-induced orientation of the azobenzene moieties confined in the SCNPs.

#### 2.1.3. Anthracene Functional Groups

**Figure 4:**
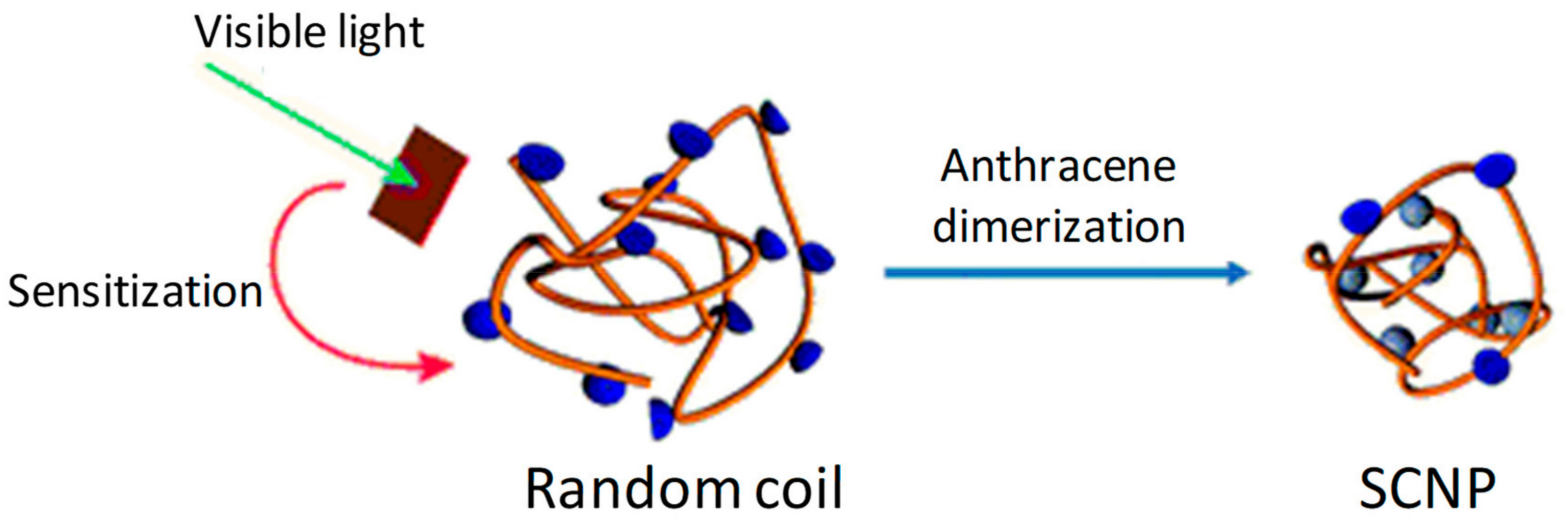
Schematic illustration of the synthesis of SCNPs through photochemical upconversion (also referred to as triplet–triplet annihilation upconversion) via intrachain dimerization of pendant anthracene groups under visible light irradiation (λ = 532 nm) in the presence of platinum octaethylporphyrin as a sensitizer. Reprinted from [31] with permission.

Berda and coworkers in 2013 pioneered the intrachain photodimerization of pendant anthracene units (Figure 2c) as an efficient route to SCNP fabrication [26]. As precursors, these authors used PMMA-based copolymers containing 10, 20 and 46 mol % of anthracene photodimerizable units. The [4 + 4] cycloaddition of pendant anthracene groups was carried out by irradiating a diluted solution of the precursor (0.1 mg/mL) with 350 nm centered UV light. The dimerization degree was determined through UV–visible spectroscopy by monitoring the disappearance of the characteristic π–π* absorption peak at ca. 360 nm from the anthracene groups. Depending on the anthracene content, different reaction times were required to ensure SCNP formation: 11.5, 22.5 and 60 min for precursors containing 10, 20 and 46 mol % of anthracene units, giving way to photodimerization degrees of 85%, 86% and 88%, respectively. SEC with triple (UV, SLS and viscosimetric) detection confirmed successful SCNP formation, although the SLS detector traces revealed the presence of a small amount of inter-chain cross-linking. The morphology of isolated SCNPs was visualized by transmission electron microscopy (TEM). In a further work by this group [27], intrachain anthracene photodimerization was used to prepare porphyrin-cored polymer nanoparticles as macromolecular models for heme iron coordination. In this case, a porphyrin core functionalized with four arms of identical polymers containing ca. 25 mol % of anthracene groups each was selected as SCNP precursor. Remarkably, the resulting nanoparticles exhibited redox and ligand-binding reactivity similar to that of native heme proteins. Subsequently, the strategy of intrachain photodimerization of pendant anthracene units was adopted by Zhao et al. to construct amphiphilic, Janus twin SCNPs [28] and biohybrid vesicles by coassembly of proteins and tadpole-shaped SCNPs [29], and by Temel and coworkers [30] to develop SCNPs as carbon nanotube catchers. On the other hand, Simon and coworkers [31] pioneered the use of visible light (λ = 532 nm) in the presence of a sensitizer (platinum octaethylporphyrin) as the driving force for SCNP formation through dimerization of pendant anthracene groups via photochemical upconversion (see Figure 4). Conventional anthracene dimerization was selected by Barner-Kowollik et al. [32] for photoactivated SCNP formation under UV irradiation at λ = 330 nm, before the photoactivated ligation of the formed SCNP with a second polymer chain using blue light (λ = 455 nm).

#### 2.1.4. Styrylpyrene Functional Groups

**Figure 5:**
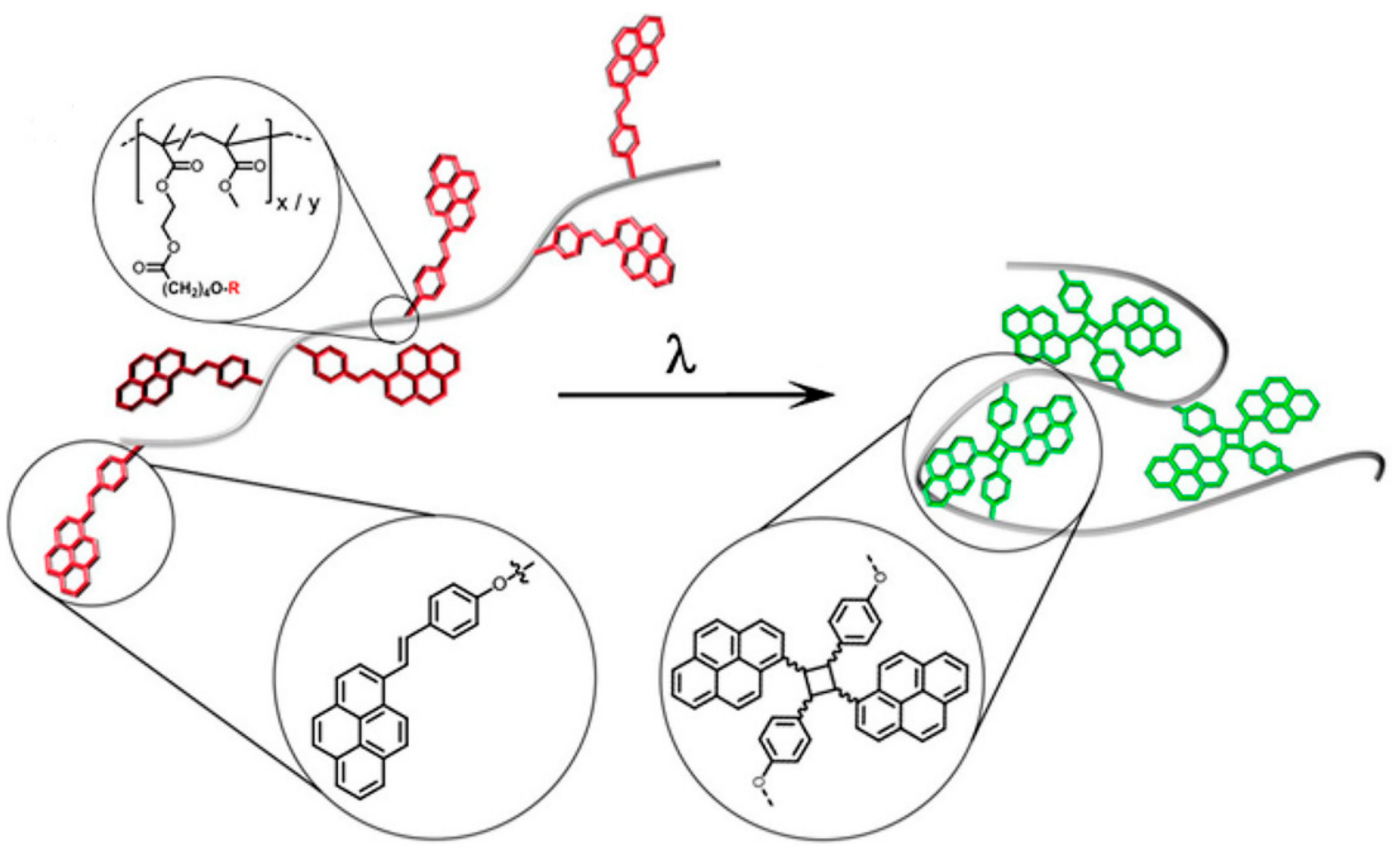
Schematic illustration of the enhanced reactivity of confined styrylpyrene moieties (shown in **red color**) within single polymer chains allowing photoactivated SCNP formation at λ = 445 nm LED-irradiation within only 15 min via single chain folding induced by formation of styrylpyrene dimers (shown in **green color**). Reprinted from [33] with permission.

Recently, Barner-Kowollik and coworkers [33] have shown in an elegant work, the significant advances offered by performing the photodimerization of styrylpyrene units (Figure 2d) in a confined macromolecular environment, such as that offered by a SCNP precursor, when compared to a control styrylpyrene [2 + 2] photocycloaddition carried out in free solution with a low-molecular-weight photoreactive compound at exactly the same absolute concentration. Hence, the enhanced reactivity of confined styrylpyrene moieties within single polymer chains allowed photoactivated SCNP formation at λ = 445 nm LED-irradiation within 15 min, or using ambient light within 24 h (see Figure 5). Critically, the advantages of photochemistry in such confined environments were a drastically increased photoreactivity toward longer wavelengths and quantum yield, when compared to a (reference) free solution environment.

#### 2.1.5. Stilbene Functional Groups

Photodimerization of stilbene units (Figure 2e) was selected by Chen and coworkers [34] to investigate the self-assembly behavior of Janus SCNPs endowed with tunable liquid crystalline properties. These Janus SCNPs were prepared via intrachain photocross-linking of stilbene-containing amphiphilic block copolymers. In this system, the size and liquid crystalline properties of the SCNPs were tuned by varying the UV irradiation time. Remarkably, upon changing the UV irradiation time and the block copolymer composition, a morphology transition from nonspherical assemblies (irregular clusters, tubular vesicles, doughnut-like assemblies and saddle-shaped lamellae) to spherical nanoparticles was observed. This transition was attributed to a significant decrease in internal liquid crystalline ordering upon increasing the UV irradiation time, as observed by small-angle X-ray scattering (SAXS) and polarized optical microscopy.

#### 2.1.6. Ketyl Radical Functional Groups

**Figure 6:**
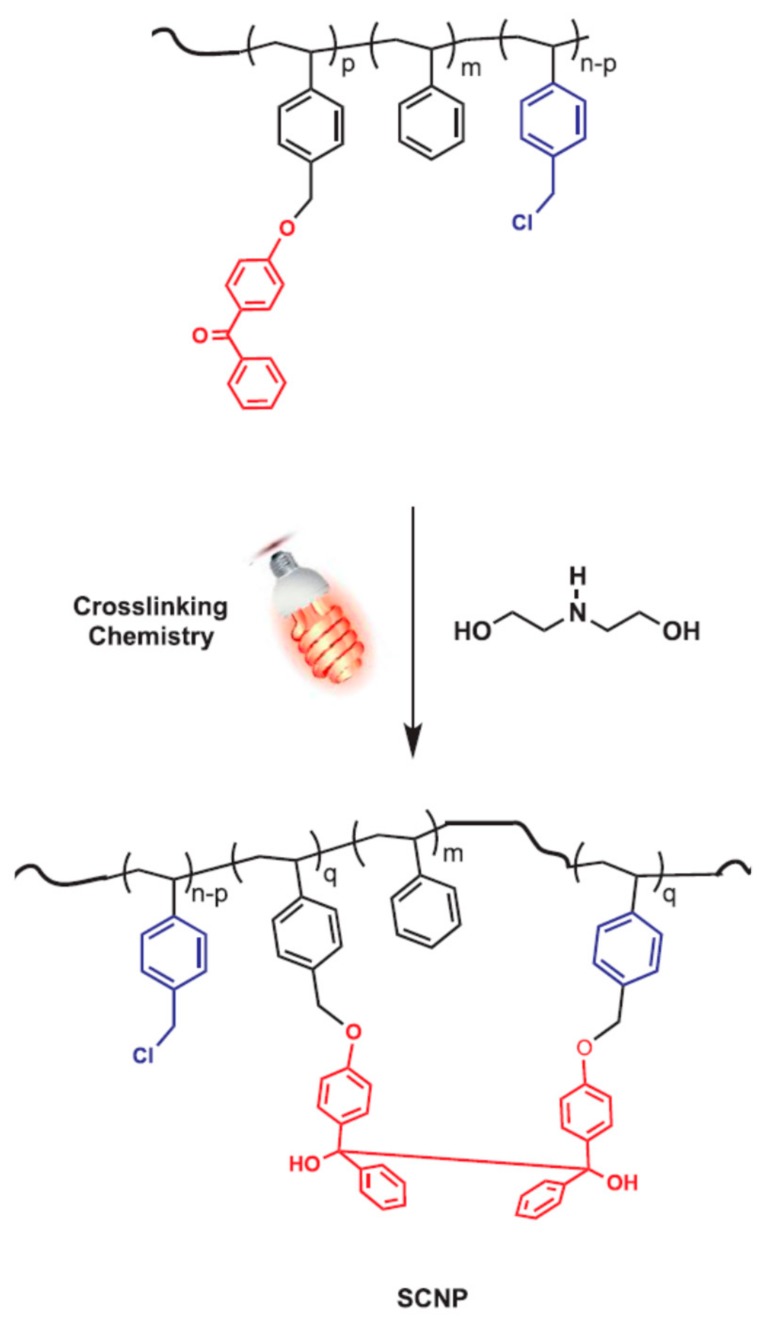
Schematic illustration of the synthesis of SCNPs through intrachain coupling of ketyl radicals from benzophenone units to give benzopinacol moieties under UV irradiation (λ = 350 nm) in the presence diethanolamine as a hydrogen donor co-initiator. Reprinted from [35] with permission.

An innovative photoactivated route toward SCNPs has been recently developed by Temel and coworkers [35], relying on the radical coupling of photogenerated ketyl radicals (see Figure 2f) from benzophenone units to give benzopinacol units after coupling. Ketyl radicals were formed through λ = 350 nm UV irradiation of copolymers containing benzophenone pendants after extensive dilution (c = 0.2 mg/mL) in acetone and in the presence of a hydrogen donor co-initiator (diethanolamine or isopropyl alcohol) (Figure 6). Successful SCNP formation after 5 h of UV irradiation was determined by means of a combination of characterization techniques involving SEC, DSC, UV and NMR spectroscopy; TEM; and atomic force microscopy (AFM) measurements.

#### 2.1.7. Carboxyl Radical Functional Groups

**Figure 7:**
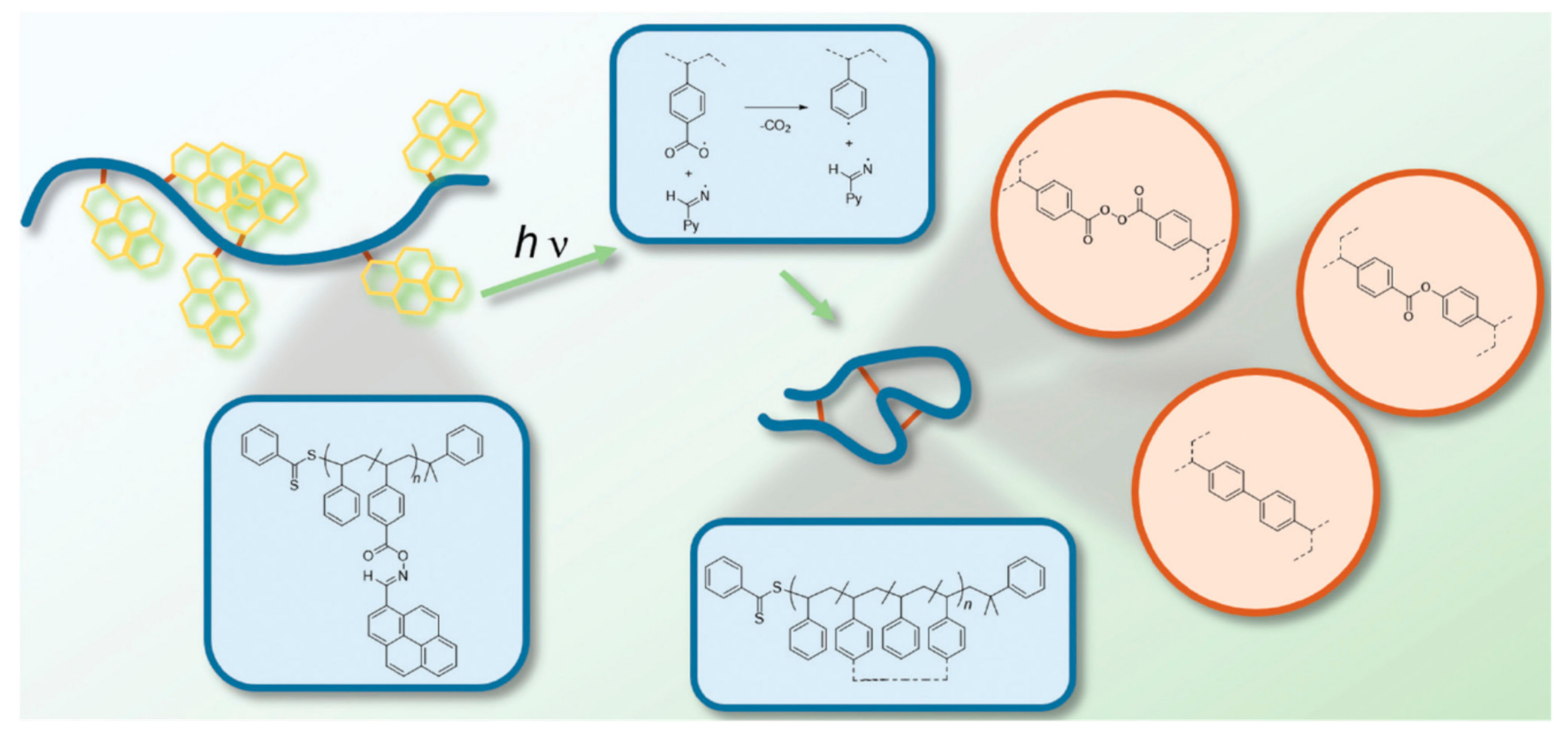
Schematic illustration of the synthesis at a high dilution of self-reporting SCNPs through intrachain coupling of radicals generated from pyrene-substituted oxime ester moieties under LED light irradiation (λ = 430–435 nm). Chain folding was accompanied by the release of pyrene units inducing fluorescence in the initially non-fluorescent solution. Reprinted from [36] with permission.

Recently, Barner-Kowollik and coworkers [36] have introduced a facile, photoinduced self-reporting methodology for the compaction of polymer chains in highly diluted solution based on intrachain radical coupling of carboxyl radicals (Figure 2g). The carboxyl radicals were generated via light-induced fragmentation of pyrene-substituted oxime ester moieties along a polymer chain. CO_2_ release from the carboxyl radicals leads to highly reactive phenyl radicals as an additional secondary species for chain compaction via homo and hetero-radical coupling events (see Figure 7). Interestingly, LED light irradiation (λ = 430–435 nm) for 2.5 h of a highly diluted solution of the SCNP precursor containing oxime ester pendants, was enough to produce SCNPs. Concomitantly, release of pyrene units after light-induced fragmentation of the oxime ester moieties allowed them to “switch on” the fluorescence of the initially non-fluorescent solution under UV irradiation at λ_exc_ = 366 nm. The resulting SCNPs were characterized in detail via SEC, UV and NMR spectroscopy measurements.

#### 2.1.8. Nitrile Imine Functional Groups

Visible light-induced nitrile imine (Figure 2h) self-dimerization has been recently employed by Barner-Kowollik et al. [37] to produce SCNPs showing fluorescent properties. Polystyrene-based polymers functionalized with pyrene-tetrazole pendants (12 mol %) were used as precursors. SCNPs were prepared upon irradiation of a very diluted solution of the precursor in dichloromethane (hydrodynamic size = 10.6 nm) with LED light (λ = 410–420 nm) for 1.5 h. Upon nitrile imine self-dimerization, the resulting SCNPs exhibited fluorescence with an emission maximum at 475 nm and a hydrodynamic size of 5.0 nm.

### 2.2. Photocyclization

**Figure 8:**
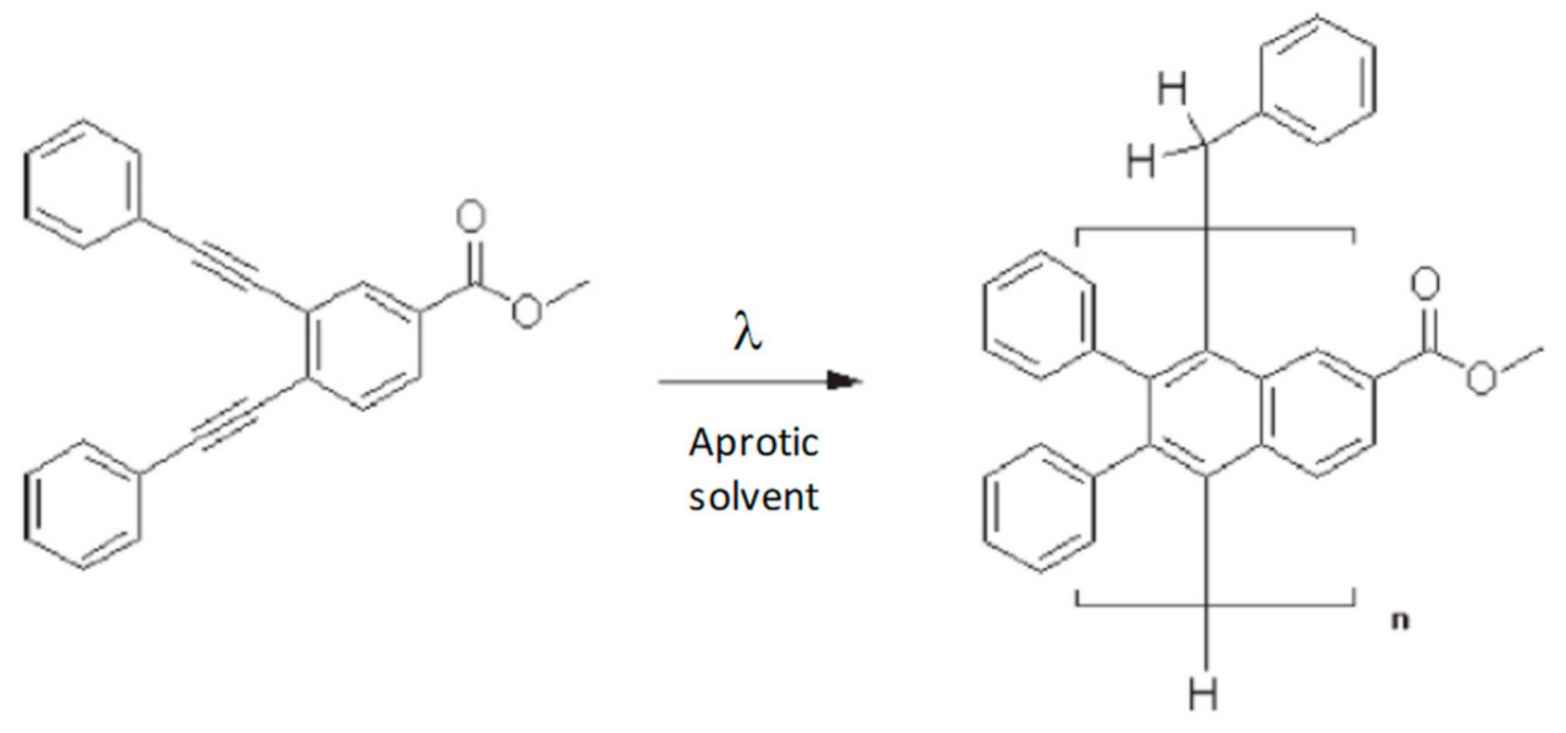
Illustration of the structure of photo-Bergman cyclization products obtained after UV irradiation of an enediyne compound in an aprotic solvent. Reprinted from [38] with permission.

Photo-Bergman cyclization was introduced by Hu and coworkers [38] as a novel yet efficient strategy to synthesize SCNPs via Bergman cyclization without the requirement of harsh thermal conditions (>200 °C). Figure 8 illustrates the chemical structure of the enediyne compound employed by these authors to give oligomeric products upon photoirradiation in aprotic solvents. Upon UV irradiation of the enediyne compound for 6 h, the presence of oligomeric products containing the naphthalene motif was revealed by NMR, Raman scattering and fluorescence spectroscopy measurements. Subsequently, SCNPs were obtained through UV irradiation for 6 h of a precursor containing very dilute, photoreactive enediyne pendants in toluene. The resulting SCNPs were characterized by Raman scattering, NMR, fluorescence spectroscopy, SEC and AFM measurements.

### 2.3. Photoisomerization

**Figure 9:**
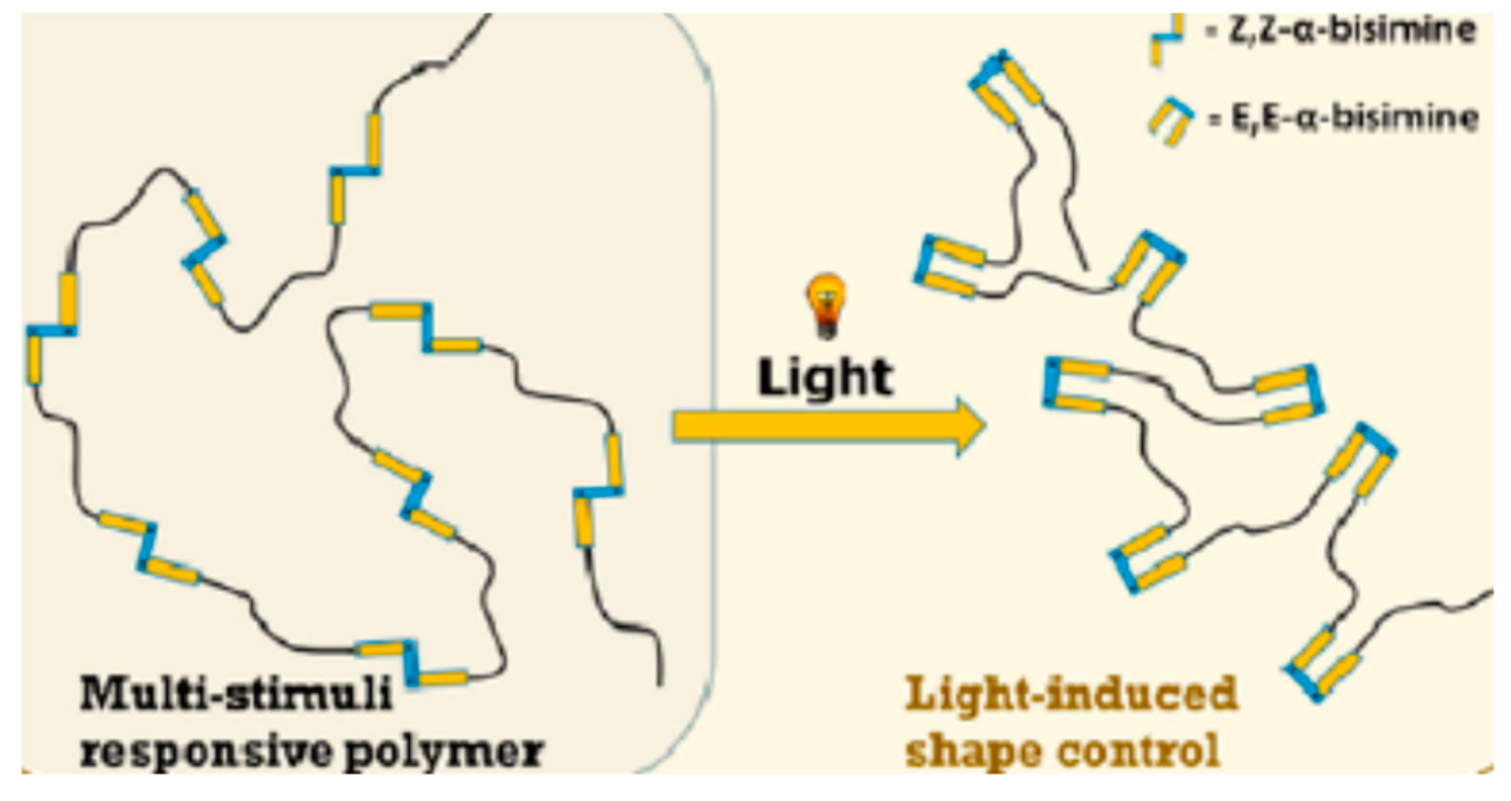
Schematic illustration of the controlled switching of size/shape with light via photoisomerization of an ADMET-polymer containing main-chain N-alkyl α-bisimine units. Reprinted from [39] with permission.

The groups of Barner-Kowollik and Lehn [39] introduced, in 2015, the use of photoresponsive N-alkyl α-bisimines as main-chain functional groups in acyclic diene metathesis (ADMET)-polymers for the controlled switching of a polymeric particle shape with light via photoisomerization (Figure 9). Upon irradiation with light of 254 nm of the (*Z/Z*)-parent precursor (hydrodynamic size = 5.1 nm), *Z/E*-photoisomerization of the C=N bond was found to give an almost pure (*E/E*)-chain (hydrodynamic size = 3.3 nm). The photoisomerization process was found to be completely reversible via a subsequent thermal back reaction (15 h, room temperature) or, alternatively, by irradiation with light of 365 nm. Remarkably, SCNP formation (hydrodynamic size = 4.3 nm) was also carried out using the same precursors via bisimine-Pd(II) complexation, conferring dual responsiveness for the controlled switching of size/shape with light and metal ions.

### 2.4. Photogeneration of Reactive Species

#### 2.4.1. Nitrenes

**Figure 10:**
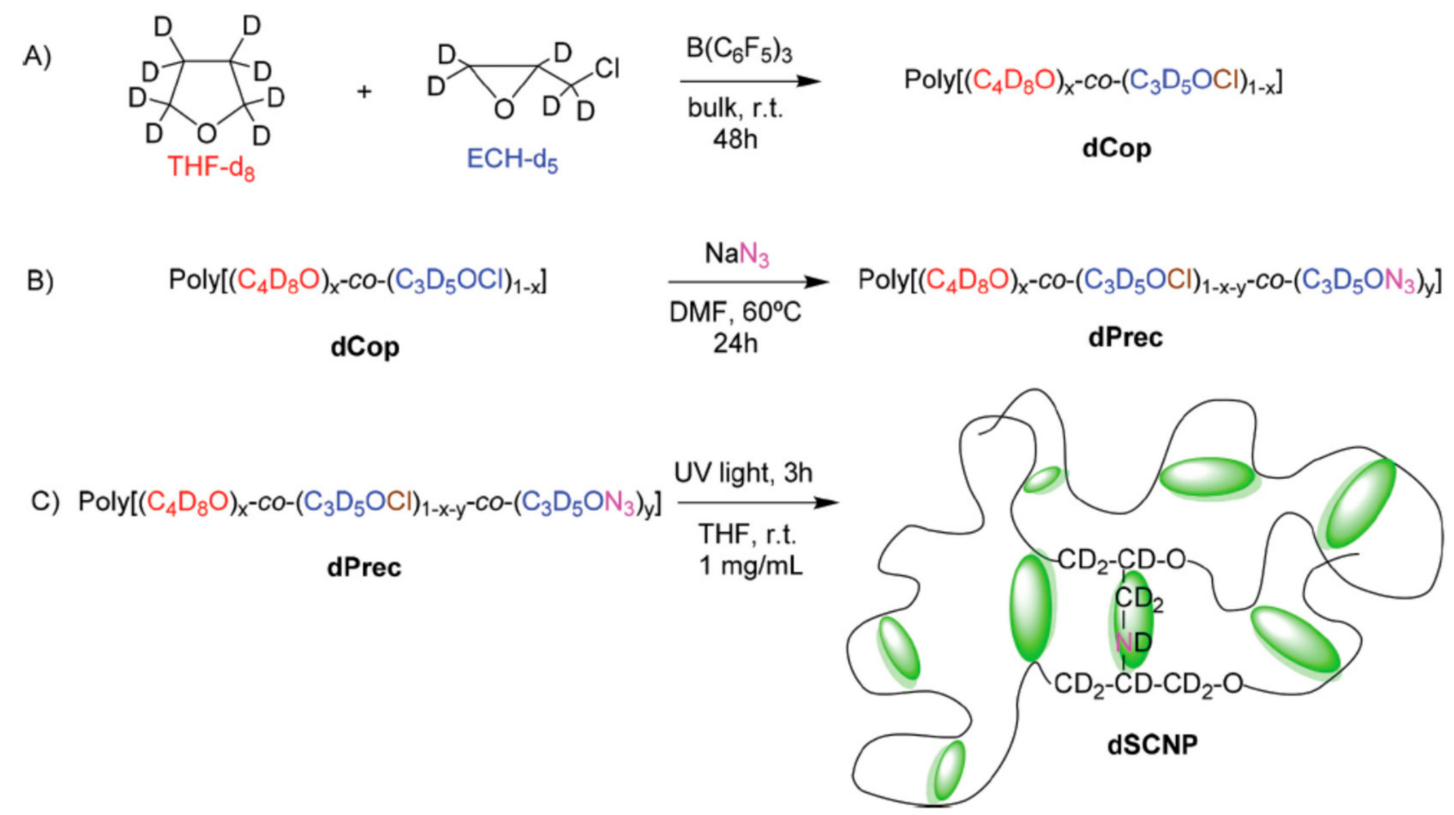
Schematic illustration of the different steps during the synthesis of completely deuterated SCNPs: (**A**) Bulk ring-opening copolymerization of available perdeuterated cyclic ether monomers. (**B**) Post-polymerization functionalization reaction with sodium azide. (**C**) SCNP formation via photogeneration of nitrene species followed by intrachain nitrene C–H insertion events. Reprinted from [41] with permission.

Bai and coworkers [40] reported, in 2014, a facile strategy for the preparation of SCNPs via intramolecular photocross-linking of azide polymers. Hence, copolymers of styrene and 4-vinylbenzyl azide containing 2.5, 5.2 and 9.6 mol % of azide groups were prepared as photoreactive SCNP precursors. Upon UV irradiation (λ = 365 nm) of the very dilute precursors in chloroform (1 mg/mL), highly reactive nitrene species formed from photodecomposition of the azide groups after release of N_2_. The folding of the linear copolymer chains to SCNPs was attributed to fast intrachain nitrene C–H insertion events. In fact, the complete disappearance of the azide moieties as followed by FTIR spectroscopy took place in only 2, 4.5 and 8 min for the copolymers containing 2.5, 5.2 and 9.6 mol % of azide groups, respectively. SEC, DLS and TEM measurements confirmed the successful formation of SCNPs. Subsequently, Pomposo and coworkers [41] adopted the technique of photogeneration of nitrene species to fabricate completely deuterated SCNPs, paving the way to deploy the full possibilities of neutron scattering techniques for investigating these soft nanomaterials. The precursors were synthetized from available perdeuterated cyclic ether monomers by a simple, bulk ring-opening copolymerization process (see Figure 10A) followed by a facile post-polymerization functionalization reaction with sodium azide as reagent (Figure 10B). UV irradiation (λ = 300–400 nm) of the very dilute precursor for 3 h produced completely deuterated SCNPs via insertion reactions upon azide decomposition (Figure 10C).

#### 2.4.2. Carbenes

More recently, De La Cuesta and Pomposo [42] produced SCNPs via photogeneration of highly reactive carbene species from copolymers containing photoreactive α-diazo-β-ketoester pendants. The efficiency of carbene generation was as high as 90.4%, as determined from elemental analysis data. SEC measurements confirmed successful SCNP formation via intrachain carbene insertion events.

### 2.5. Self-Assembly Induced by Photodeprotection

#### 2.5.1. Hydrogen-Bonded Dimers

**Figure 11:**
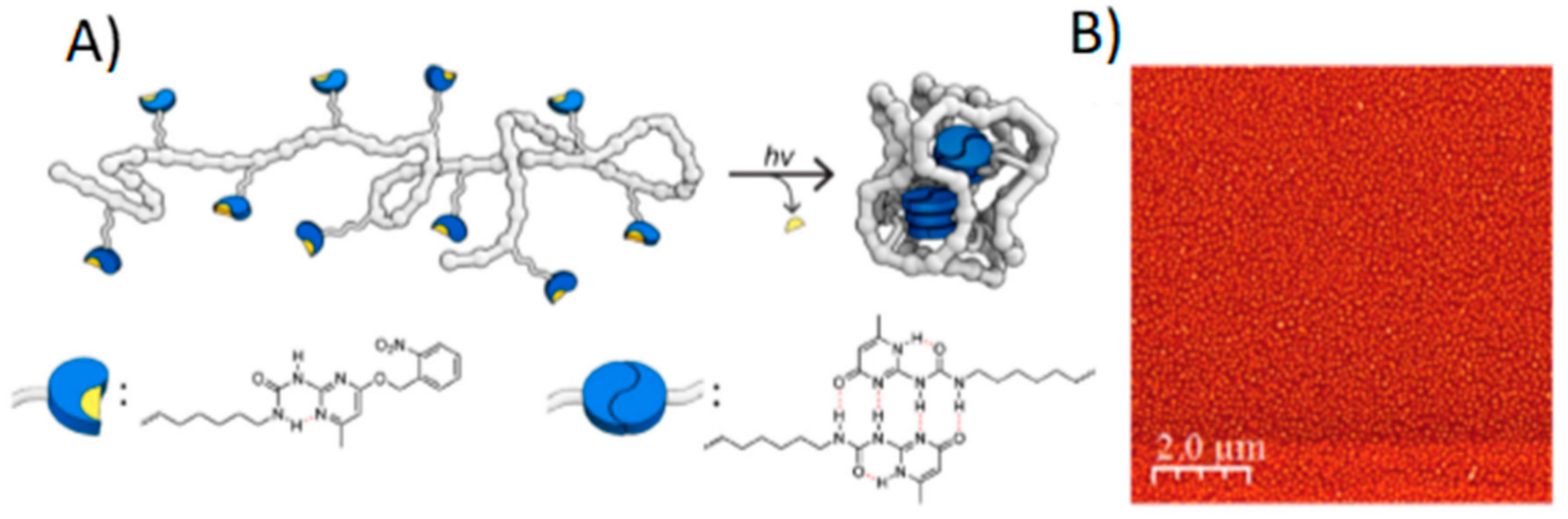
Schematic illustration of the synthesis of reversible, non-covalent bonded SCNPs via photodeprotection of ο-nitrobenzyl-substituted 2-ureido-pyrimidinone (UPy) moieties, followed by UPy dimerization via quadruple hydrogen bonding interactions, (**A**) and an AFM picture of the resulting SCNPs (**B**). Reprinted from [44] with permission.

Meijer and coworkers, in 2009, pioneered the synthesis of reversible, non-covalent bonded SCNPs via photodeprotection of *o*-nitrobenzyl-substituted 2-ureido-pyrimidinone (UPy) moieties [43,44]. Polymers decorated with *o*-nitrobenzyl-protected UPy pendants were selected as precursors since the bulky protecting group prevented UPy dimerization via quadruple hydrogen bonding interactions; hence, SCNP formation. UV irradiation (λ = 350 nm) of a highly diluted solution of the precursors in chloroform resulted in removal of the protecting groups and SCNP formation, as determined by SEC and AFM measurements (Figure 11). The reversible nature of the SCNPs was revealed by adding formic acid to the SCNP solution that disrupted the hydrogen bonding interactions leading to a hydrodynamic size of the chains similar to that of the initial precursor. This group further used the strategy of dimerization induced by photodeprotection to establish useful structure–function relationships in non-covalently bonded SCNPs, as summarized in a recent review paper [44].

#### 2.5.2. Hydrogen-Bonded Helical Stacks

**Figure 12:**
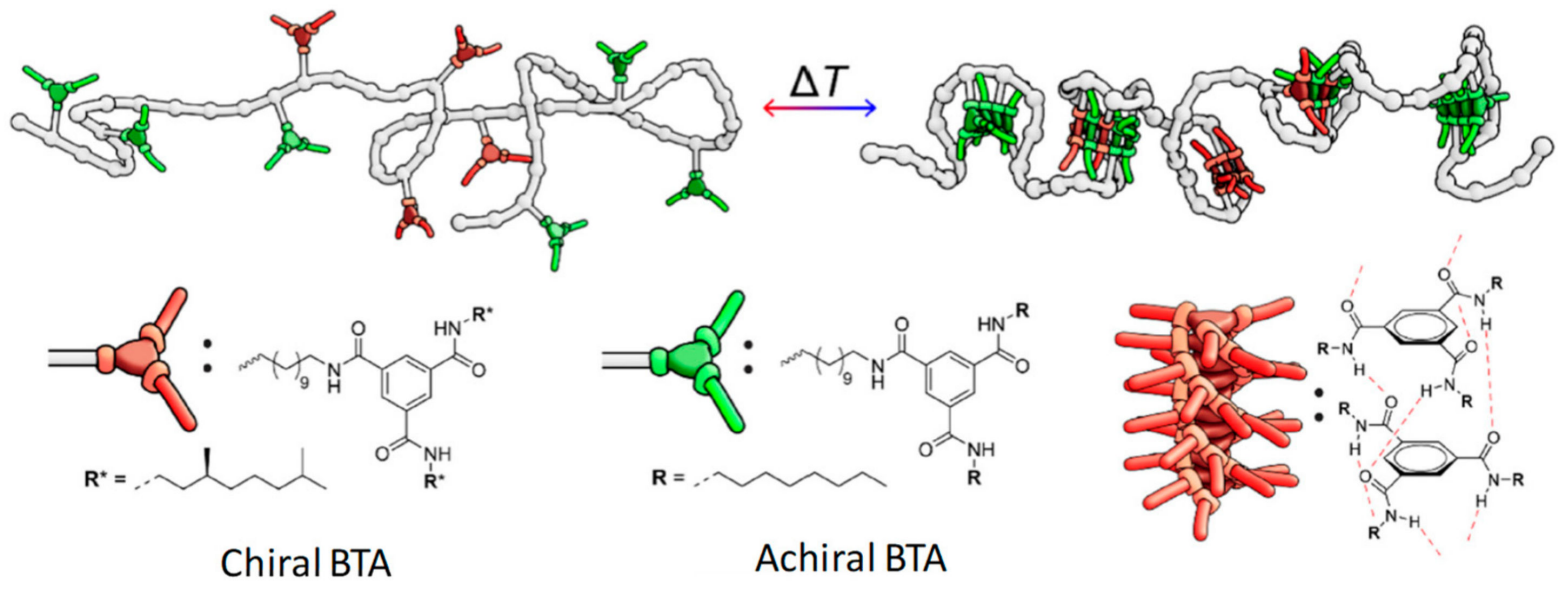
Synthesis of reversible, non-covalent bonded SCNPs via helical stacking using a precursor containing both chiral (in **orange color**) and achiral (in **red color**) benzene-1,3,5-tricarboxamide (BTA) units showing the formation of multiple, segregated BTA stacks instead of a single chiral BTA stack. Reprinted from [44] with permission.

Subsequently, Palmans, Meijer and coworkers, in 2011, were the first to investigate the preparation of reversible, non-covalent bonded SCNPs via helical stacking induced by photodeprotection [45]. As photoprotected functional groups, they selected *o*-nitrobenzyl-substituted benzene-1,3,5-tricarboxamide (BTA) moieties which were attached to linear poly(isobornyl methacrylate) chains by means of a postfunctionalization approach. The presence of the bulky *o*-nitrobenzyl-protecting group prevented the BTAs of the precursor to self-assemble into helical stacks stabilized by three-fold hydrogen-bonding; hence, SCNP formation. Interestingly, step-wise photodeprotection of the *o*-nitrobenzyl-substituted BTAs (UV light, λ = 350 nm) induced the folding of the precursor in a step-wise manner. Circular dichroism (CD) spectroscopy revealed that the BTAs did still self-assemble into helical stacks in the SCNPs. Several different supramolecular SCNPs based on helical stacking (see Figure 12) are the subject of a recent review paper by this group [44].

## 3. Synthesis of Single-Chain Polymer Nanoparticles via Phototriggered Intrachain Heterocoupling

Phototriggered intrachain heterocoupling refers to the folding/collapse process induced by photons to an isolated polymer chain containing complementary (hetero)functional groups (i.e., A-type and B-type) that react between them, upon photoactivation, in a pairwise manner (i.e., A + B) (see Scheme 1B).

### 3.1. Photoinduced Diels–Alder (DA) Reaction

**Figure 13:**
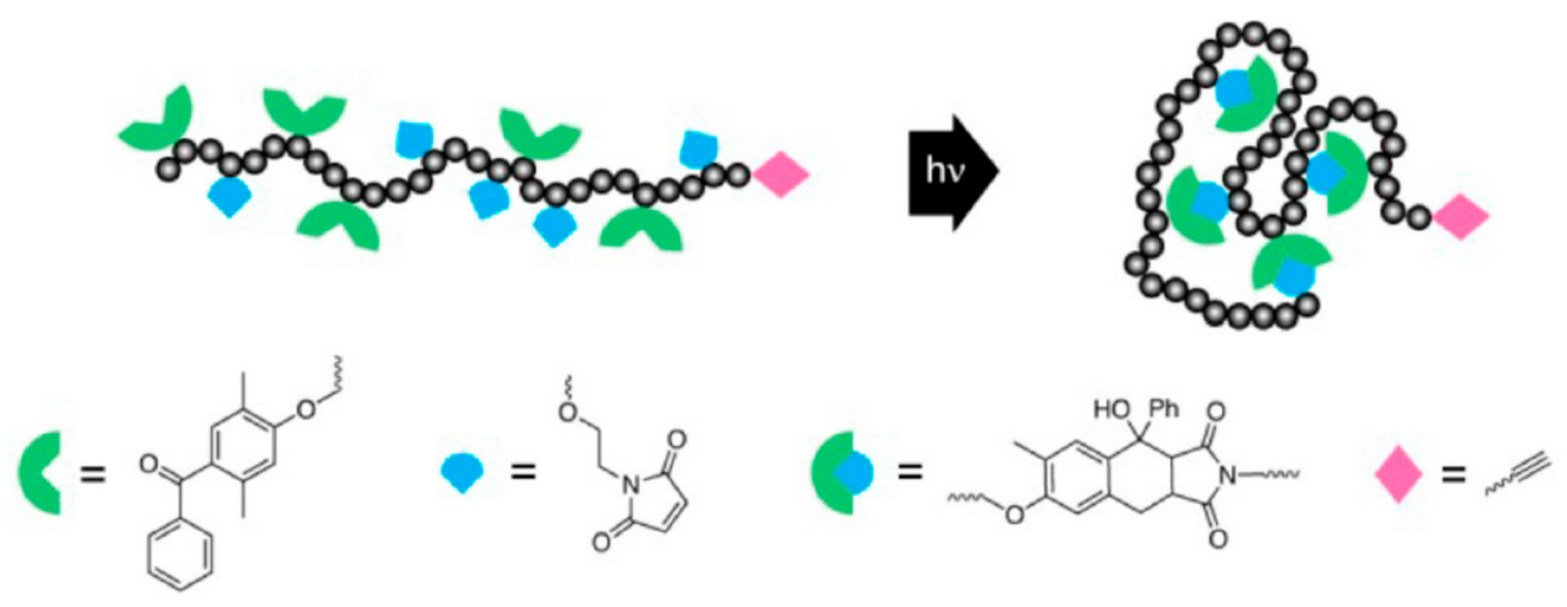
Functional SCNP formation via photoinduced Diels–Alder (DA) ligation using a precursor having 2,5-dimethyl benzophenone (in **green color**) and *N*-maleimide pendants (in **blue color**), and an alkyne functional group as the chain-end (in **pink color**). Reprinted from [46] with permission.

Barner-Kowollik and coworkers introduced, in 2013, the UV-light-triggered DA ligation as a mild and efficient approach to produce functional SCNPs [46]. Precursors were prepared based on polystyrene copolymers containing 2,5-dimethyl benzophenone (DMBP) and *N*-maleimide (Mal) pendants and an alkyne functional group as the chain-end. Upon UV irradiation (λ = 320 nm) for 30 min of a diluted solution of the precursor (0.017 mg/mL), the photoactivated DMBP groups were found to react intramolecularly with the Mal units via DA ligation resulting in the formation of SCNPs through intrachain heterocoupling (Figure 13). The resulting functional SCNPs were characterized by SEC, NMR, DLS and AFM measurements. In a further work, a functionalized polythiophene-containing DMBP and Mal groups was used to produce π-conjugated SCNPs via photoinduced DA reaction [47].

### 3.2. Photoinduced Nitrile Imine-Mediated Tetrazole-ene Cycloaddition (NITEC)

**Figure 14:**
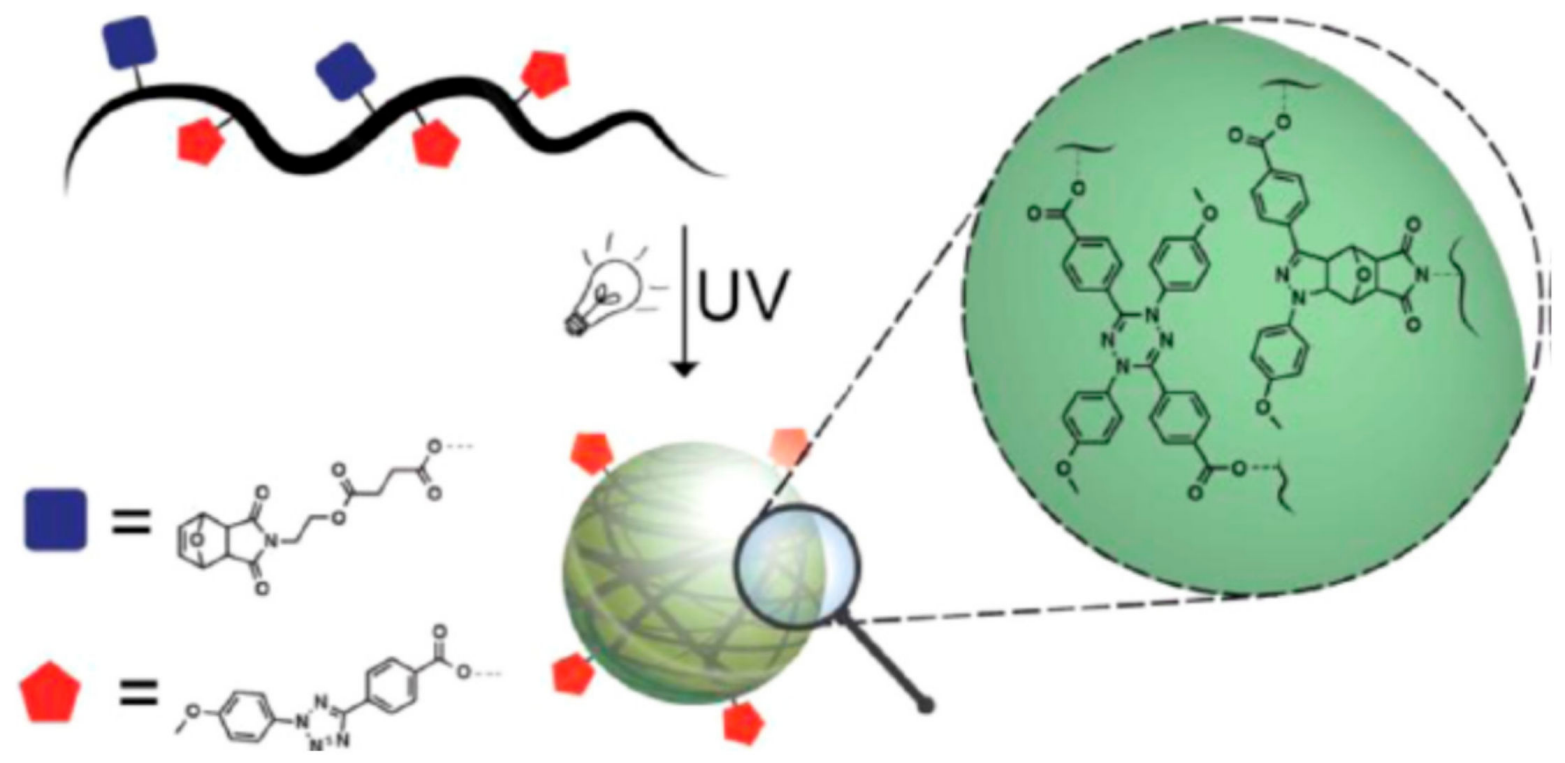
SCNP formation via photoinduced NITEC using polymers containing protected maleimide (in **blue color**) and tetrazole (in **red color**) moieties. In addition to the NITEC heterocoupling reaction, the nitrile imine self-dimerization from tetrazole units (homocoupling reaction) takes place as a secondary reaction. Reprinted from [48] with permission.

In another contribution by the Barner-Kowollik group [48], the phototriggered intrachain heterocoupling for SCNP formation relied on photoinduced nitrile imine mediated tetrazole-ene cycloaddition (NITEC) by using polymers containing protected maleimide (PG-Mal) and tetrazole (Tet) moieties (Figure 14). Remarkably, the resulting SCNPs were endowed with fluorescent properties due to the fluorescence of the pyrazoline cycloadducts formed upon NITEC. Hence, a broad fluorescence band centered at 558 nm was observed upon excitation of the SCNPs at λ_exc_ = 415 nm. Fluorescence was also observed in the case of SCNPs obtained via photoinduced NITEC using polybutadiene [49] and glycopolymer [50] precursors both decorated with PG-Mal and Tet units.

### 3.3. Photoinduced Nitrile Imine–Carboxylic Acid Ligation (NICAL)

Similar to the case of photoinduced NITEC, appropriate copolymers containing Tet as well as carboxylic acid moieties were collapsed, with success, to SCNPs by Barner-Kowollik and coworkers via photoinduced, intrachain nitrile imine–carboxylic acid ligation (NICAL) [51].

## 4. Synthesis of Single-Chain Polymer Nanoparticles via Photogenerated Collapse Induced by External Cross-Linkers

**Figure 15:**
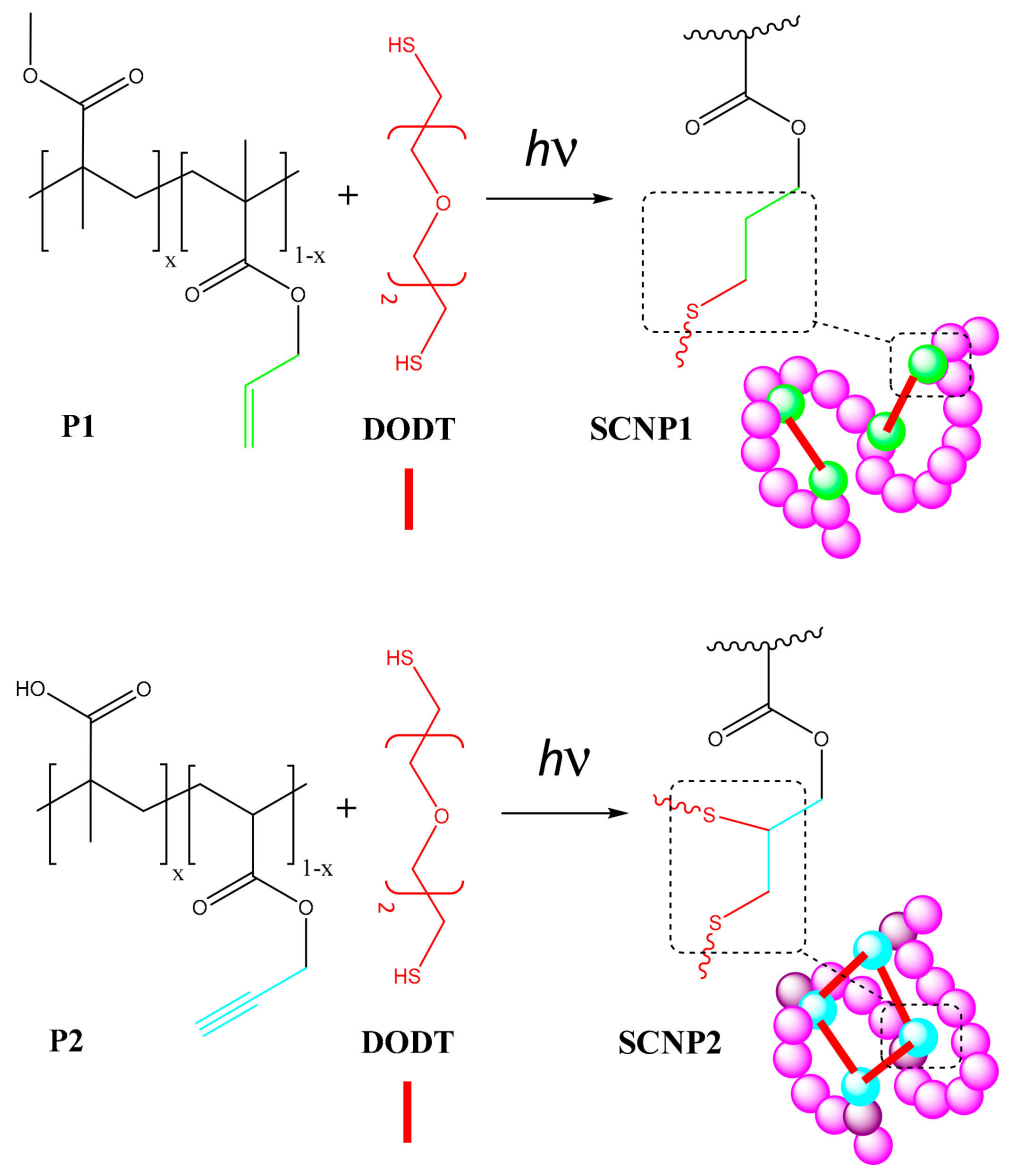
Synthesis of SCNPs via photoactivated radical-mediated thiol-ene/thiol-yne coupling using 3,6-dioxa-1,8-octane-dithiol (DODT) as a homobifunctional cross-linker (in **red color**) and copolymers **P1** and **P2** as precursors decorated with alkene (in **green color**) and alkyne (in **blue color**) pendants, respectively. Reprinted from [52] with permission.

Pomposo and coworkers reported, in 2014, the room temperature synthesis of SCNPs via photoactivated radical-mediated thiol-ene/thiol-yne coupling by using 3,6-dioxa-1,8-octane-dithiol (DODT) as a homobifunctional cross-linker (see Scheme 1C) [52]. As precursors, copolymers decorated with alkene or alkyne pendants synthesized via redox-initiated RAFT polymerization were used (see Figure 15). Remarkably, higher chain compaction was observed in the case of SCNPs prepared via photoactivated radical-mediated thiol-yne coupling, as observed by SAXS measurements and supported by molecular dynamics (MD) simulations. In a subsequent work [53], the merging of zwitterionic ring opening polymerization (ZROP) and photoactivated radical-mediated thiol-yne coupling allowed, for the first time, the synthesis of elusive polyether SCNPs, broadening the scope of photoactivated radical-mediated thiol-yne coupling for SCNP formation. Additionally, photogenerated collapse induced by UV irradiation of external bis-azide-containing aggregation induced emission (AIE) cross-linkers has been employed by De La Cuesta and Pomposo [42] as a fast and efficient method to produce highly fluorescent SCNPs. Recently, SCNPs have been additionally prepared through photogenerated collapse using external cross-linkers via photoinduced NICAL [37] and via photoinduced nitroxide radical-α-hydroxyalkyl phenyl ketone coupling [54].

## 5. Synthesis of Single-Chain Polymer Nanoparticles via Photoactivated Multifolding

### 5.1. Simultaneous Photoactivated Multifolding

**Figure 16:**
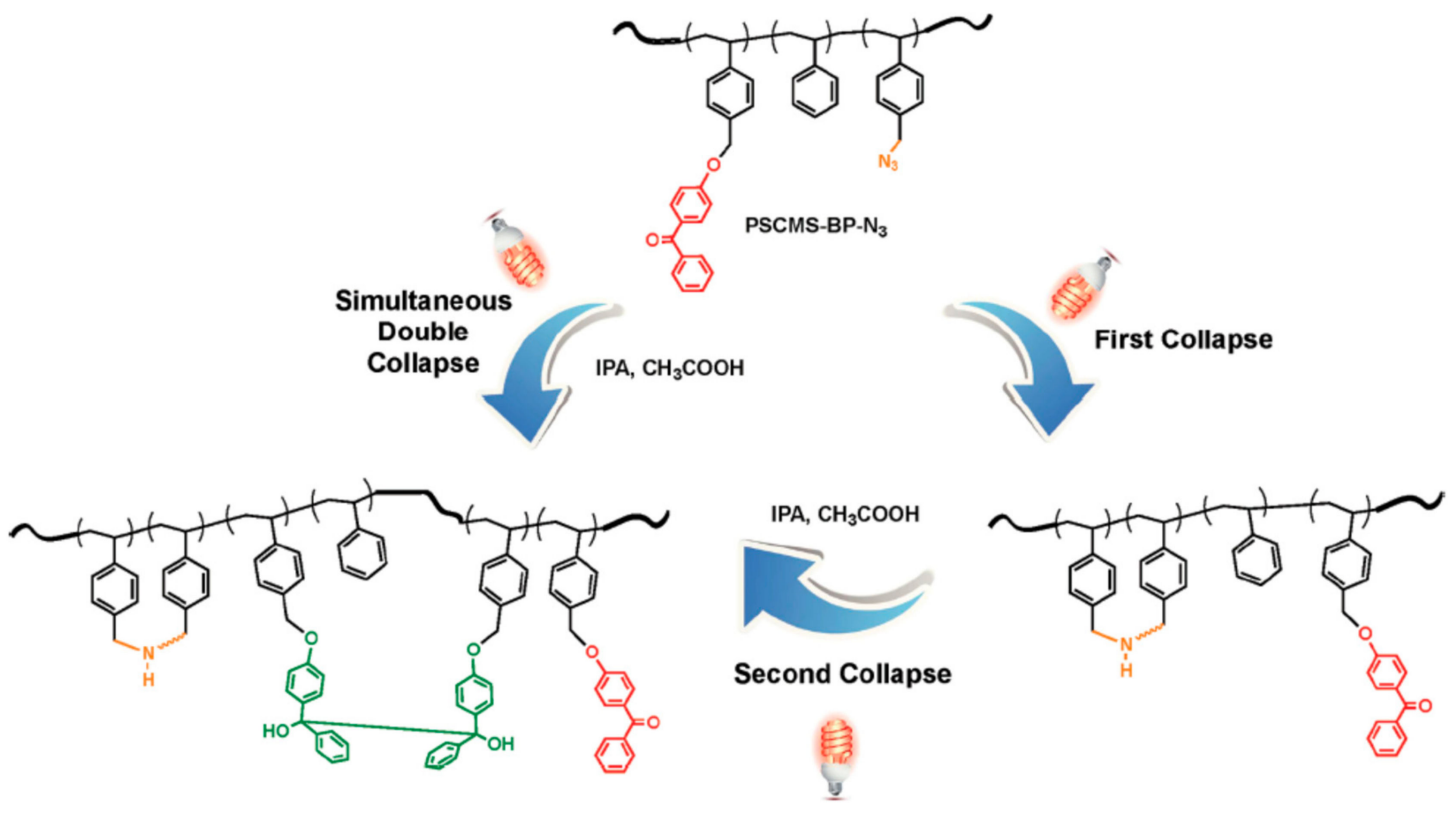
Illustration of SCNP formation via simultaneous (**left**) or sequential (**right**) photoactivated double folding/collapse. IPA = isopropyl alcohol. Reprinted from [55] with permission.

Barner-Kowollik and coworkers reported simultaneous, photoactivated multifolding via photoinduced NITEC and nitrile imine self-dimerization in copolymers containing protected maleimide and tetrazole units [48,51]. This group also pioneered the use of simultaneous photoactivated NITEC and NICAL to induce the folding of amphiphilic precursors to fluorescent SCNPs in pure water [50]. More recently, Temel and coworkers [55] have introduced a straightforward method to produce SCNPs by the simultaneous, photoactivated (UV light, λ = 350 nm) double folding of single linear chains containing both pendant azide and benzophenone motifs (see Figure 16-left). Interestingly, these authors performed a clear comparison between SCNPs prepared through simultaneous against sequential, photoactivated double folding (see next section).

### 5.2. Sequential Photoactivated Multifolding

#### 5.2.1. Without External Cross-Linkers

**Figure 17:**
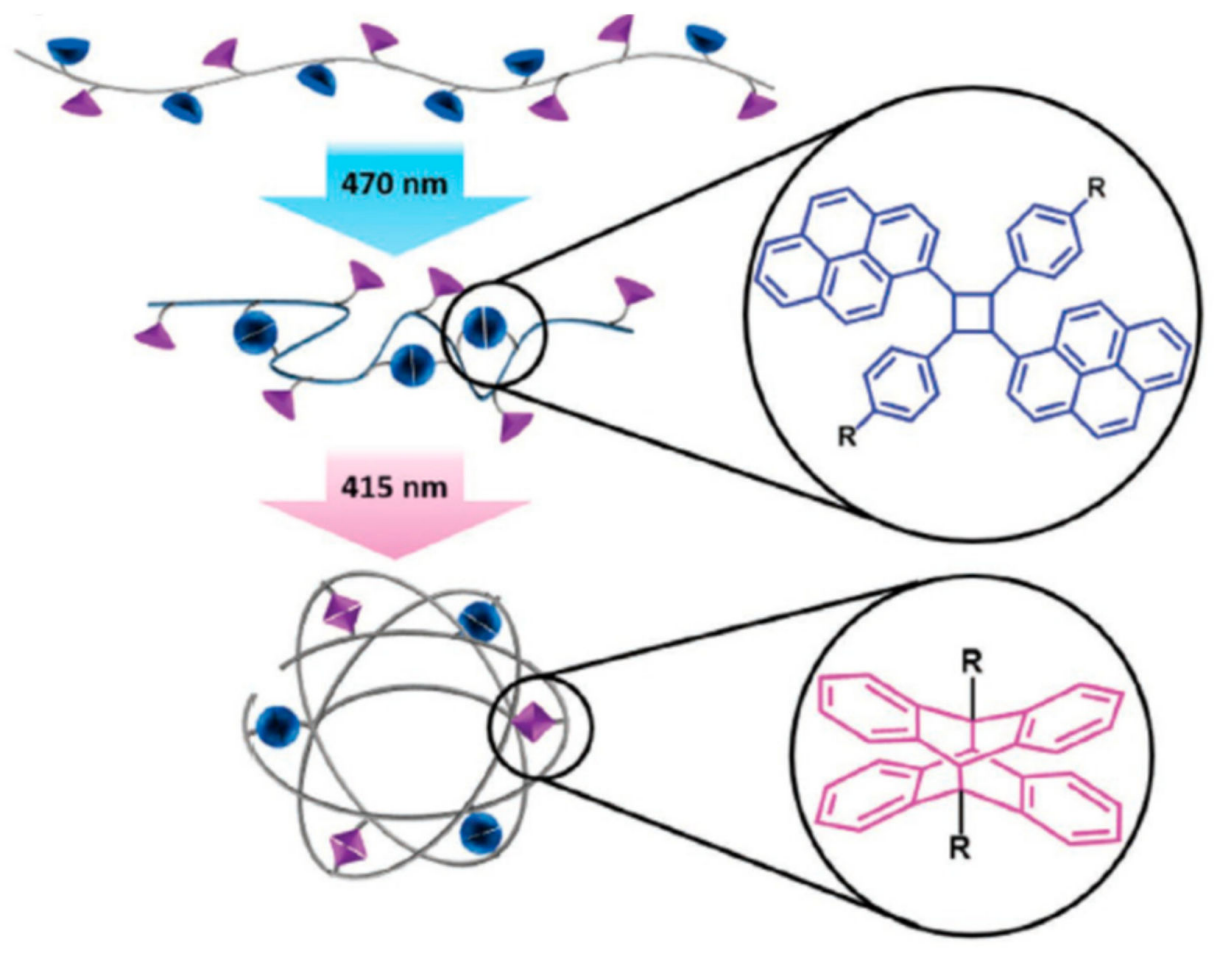
A schematic illustration of wavelength-selective folding of single polymer chains decorated with styrylpyrene and anthracene groups with different colors of visible light: irradiation with blue light (λ = 470 nm) triggered the [2 + 2] photocycloaddition of styrylpyrene units as a first step, whereas violet light (λ = 415 nm) induced the [4 + 4] photocycloaddition of anthracene groups as a second step. Reprinted from [56] with permission.

Figure 16-right shows SCNP fabrication using the sequential, photoactivated double folding of a precursor containing azide and benzophenone moieties, as recently reported by Temel and coworkers [55]. Intrachain nitrene C–H insertion upon azide decomposition was selected as the first folding process. The second folding process relied on intramolecular benzopinacol formation in the presence of isopropyl alcohol (IPA) as a hydrogen donor. A comparison of SCNPs prepared by these authors via simultaneous versus sequential photoactivated double folding revealed similar values of apparent molecular weight (5630 versus 5400 g/mol), hydrodynamic radius (6.6 versus 6.9 nm) and glass transition temperature (151 versus 150 °C). Subsequently, Barner-Kowollik et al. [56] reported the wavelength-selective folding of single polymer chains decorated with styrylpyrene and anthracene groups with different colors of visible light. In a first step, irradiation with blue light (λ = 470 nm) triggered the [2 + 2] photocycloaddition of styrylpyrene units, whereas violet light (λ = 415 nm) induced the [4 + 4] photocycloaddition of anthracene groups in a subsequent step (see Figure 17).

#### 5.2.2. With External Cross-Linkers

**Figure 18:**
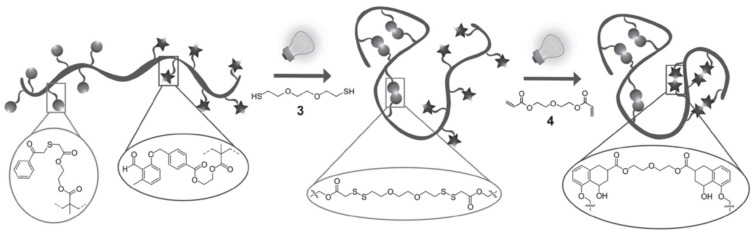
Schematic illustration of the sequential photoactivated multifolding of a precursor containing phenacyl sulfide and protected α-methylbenzaldehyde units with a dithiol cross-linker (λ = 355 nm, 30 min) and a diacrylate cross-linker (λ = 320 nm, 30 min), respectively. Reprinted from [57] with permission.

Pioneering work by Perrier, Delaittre, Barner-Kowollik and coworkers [57] showed the stepwise, e light-induced dual compaction of single polymer precursors to SCNPs assisted by the use of external cross-linkers in each folding step (Figure 18). As precursors, block copolymers containing phenacyl sulfide and protected α-methylbenzaldehyde pendants were selected. The first folding step was activated with irradiation at 355 nm in the presence of a free dithiol cross-linker for 30 min. The second folding process was performed by irradiation at 320 nm for 30 min using a flexible, diacrylate cross-linker. At least for this system, SEC and DLS showed a significantly higher degrees of chain compaction in the first step than in the second one.

## 6. Phototriggered Disassembly of Single-Chain Polymer Nanoparticles

### 6.1. Main-Chain Disassembly

**Figure 19:**
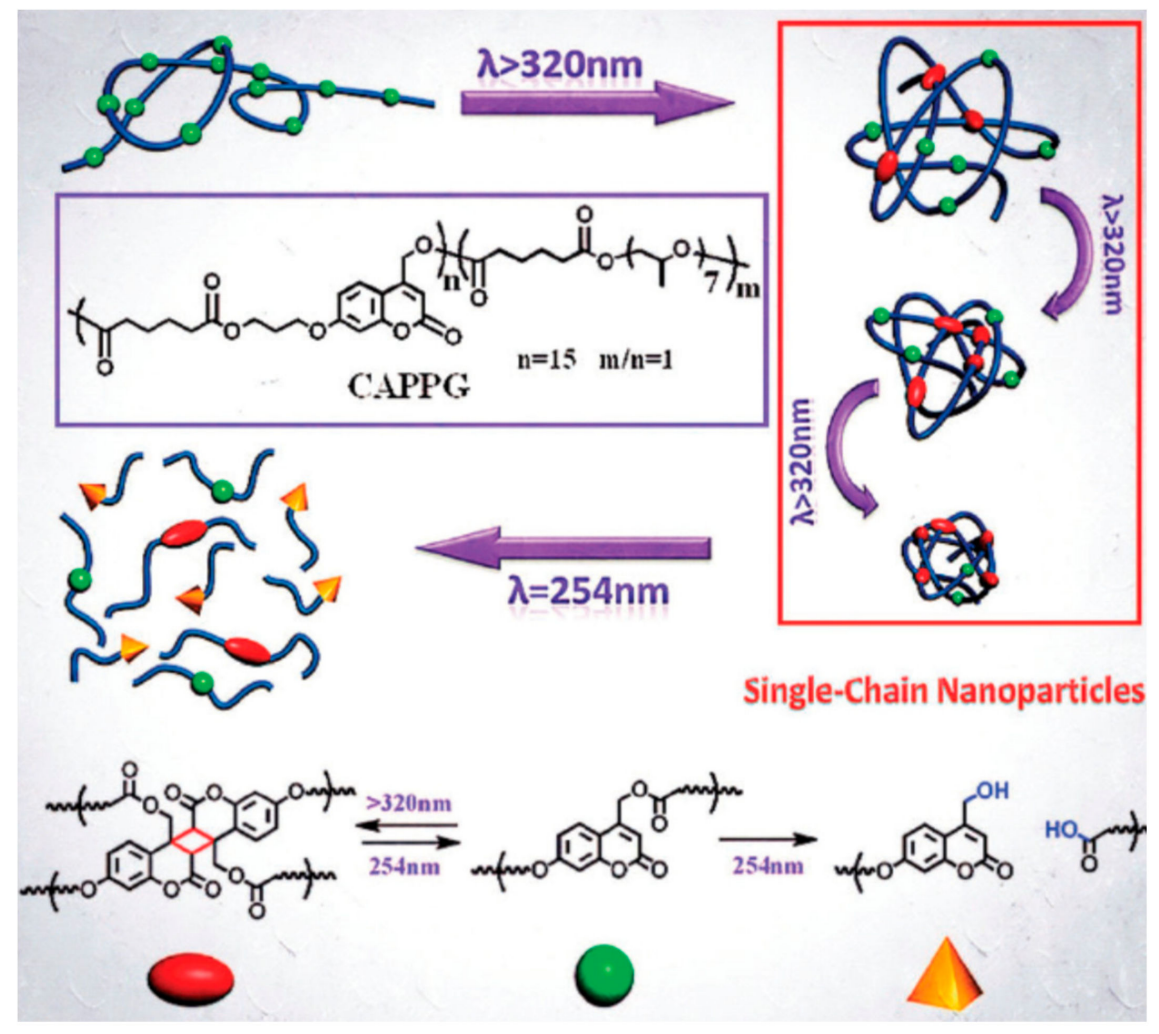
Schematic illustration of the formation of size-tunable SCNPs from a polyester precursor bearing coumarin moieties in the main chain (in **green color**) via UV-irradiation (λ > 320 nm) of a very dilute solution to give coumarin dimers (in **red color**) and subsequent main-chain disassembly through UV-irradiation at λ > 254 nm, leading to coumarin-photocleaved fragments (in **yellow color**). Reprinted from [58] with permission.

In a pioneering work, Zhao and coworkers [58] reported the preparation of size-tunable and photodegradable SCNPs using a polyester precursor (hydrodynamic radius = 5.2 nm) bearing coumarin moieties in the main chain (see Figure 19). Hence, SCNPs resulted upon UV irradiation at λ > 320 nm of this precursor under very diluted conditions. SEC and DLS measurements revealed that SCNP size decreases upon increasing UV irradiation time. The degree of coumarin dimerization was found to reach a maximum level of 87% after 2 h of UV irradiation at λ > 320 nm (SCNP hydrodynamic radius = 3.0 nm). Remarkably, photo-triggered disassembly of the SCNPs was observed upon progressive UV-irradiation at λ > 254 nm (up to a limiting time of 3 h), as determined by SEC and DLS measurements. SCNP disassembly was attributed to photocleavage of the coumarin chromophores under such conditions (see Figure 19).

### 6.2. External Cross-Linker Disassembly

**Figure 20:**
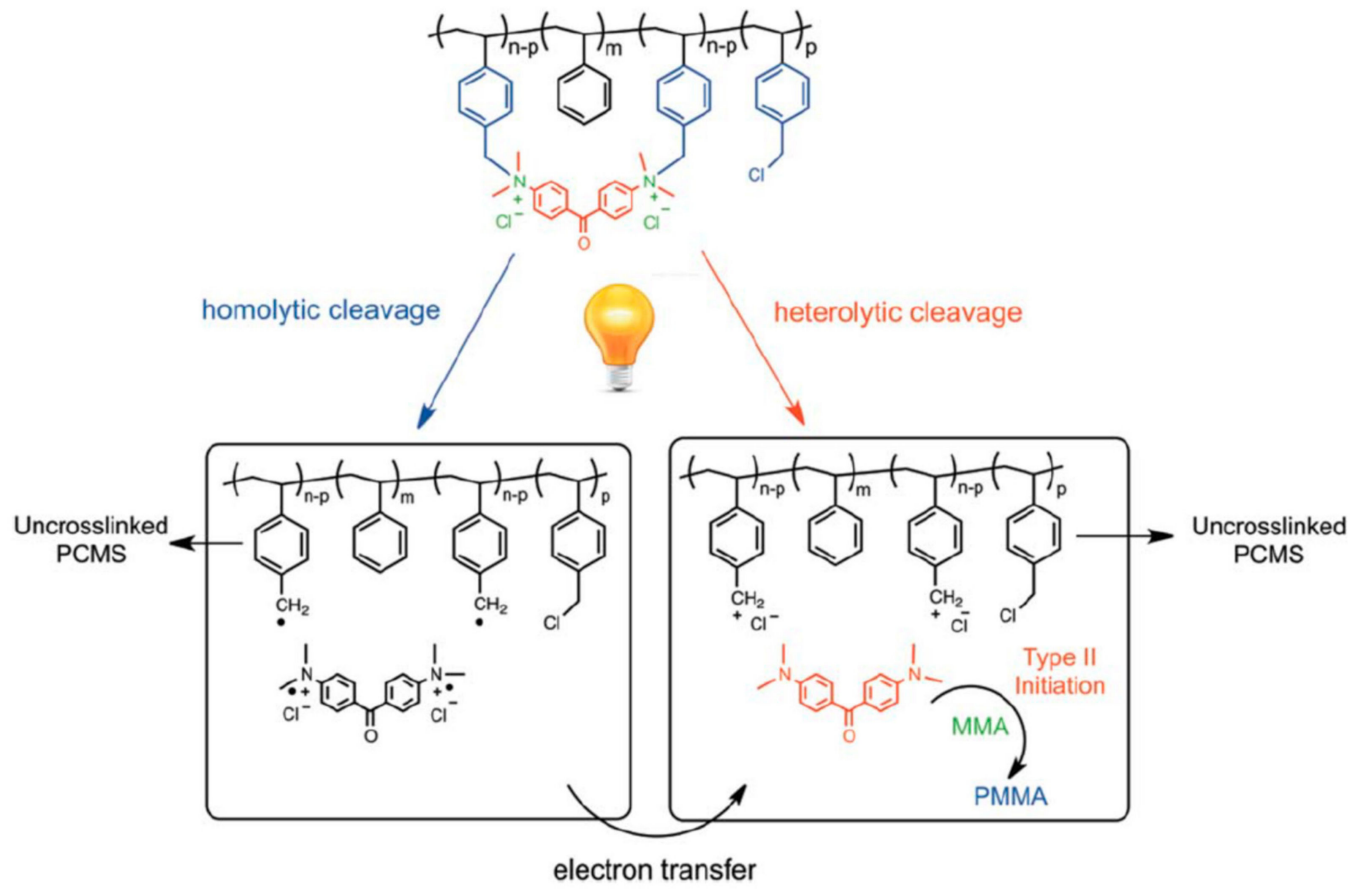
General mechanism of unfolding of SCNPs via phototriggered external cross-linker disassembly for SCNPs prepared from a polystyrene copolymer containing –CH_2_–Cl pendants (PCMS) using Michler′s ketone as an external cross-linker, and concurrent photopolymerization of an MMA monomer by the released Michler′s ketone units. Reprinted from [59] with permission.

Recently, Temel and coworkers [59] have reported the unfolding of SCNPs via phototriggered external cross-linker disassembly (Figure 20). First, SCNPs were synthesized at with a very dilute solution via Menschutkin chemistry from a polystyrene copolymer containing –CH_2_–Cl functional groups using Michler′s ketone (4,4′-bis(dimethylamino)benzophenone) as an external cross-linker. Successful SCNP formation was certified by means of a combination of techniques, including SEC, DLS, FTIR, DSC and TEM. Remarkably, it was found that the Michler′s ketone parts of the SCNPs were highly reactive under photoexcitation, so UV irradiated SCNPs (λ = 350 nm, 2 h, air atmosphere) were able to initiate methyl methacrylate (MMA) polymerization without the need for an external initiator. Concurrently to the release of Michler´s ketone upon UV irradiation, unfolding of the SCNPs was observed through SEC and DLS measurements.

## 7. Applications of SCNPs Prepared from Photocross-linking

Several applications have been suggested for the different SCNPs prepared from photocross-linking, including (i) imprintable nano-materials with high binding capacities and selectivities [21]; (ii) tunable nanoreactors for the synthesis of gold nanoparticles (AuNPs) [23,24]; (iii) CO_2_-capture nanomaterials [24]; (iv) advanced Janus surfactants [28]; (v) biohybrid vesicles as protein carriers [29]; (vi) carbon nanotube catchers [30]; (vii) photoresponsive liquid crystalline nanomaterials [25,34]; (viii) new, fluorescent, soft nanomaterials [26,27,28,29,30,31,32,36,37,38,40,42,47,48,49,50,53]; (ix) adaptative, dynamic nano-particles [43,44,45]; (x) totally deuterated nanomaterials for the investigation of the structures and dynamics of all-polymer nanocomposites [41]; (xi) photodegradable nanomaterials [58]; and (xii) nanoreactors for the syntheses of polymers [59].

## 8. Conclusions

Clean use of photons from light to activate chemical reactions offer many possibilities for the phototriggered synthesis of SCNPs as soft nanomaterials with promising applications in enzyme-mimicking catalysis and nanomedicine, among other different uses. As illustrated in this review article, the energy of photons in the ultraviolet (UV) and visible regions of the electromagnetic spectrum is enough to promote a large variety of photoreactive moieties to excited electronic states. Hence, activation of phototriggable functional groups in a SCNP precursor copolymer under highly diluted conditions to drive subsequent intrachain reactions opens the door to facile SCNP fabrication under very mild photochemical conditions, without the use of high temperatures or aggressive reagents. However, in some cases, intra-chain photocross-linking could be accompanied by secondary reactions, such as chain scission events. Consequently, it is always necessary to check carefully whether true SCNP formation is taking place, and the extension of competitive reactions. Nowadays, the different strategies developed for the phototriggered synthesis of SCNPs are: (i) photoactivated intrachain homocoupling, with a variety of photodimerizable moieties available, such as cinnamoyl, coumarin, anthracene, styrylpyrene, stilbene, ketyl radical, carboxyl radical and nitrile imine functional groups; (ii) phototriggered intrachain heterocoupling; and (iii) photogenerated collapse induced by an external cross-linker. The current research interest is shifting towards visible-light photoactivated multifolding applied to SCNP construction, and innovative strategies for the phototriggered disassembly of SCNPs. In our opinion, this emerging field will continue to evolve towards increased precision of SCNP assembly and disassembly, as well as towards broader applications, including biomedical ones. Advances on all of these topics are expected in the coming years.
